# Ammonia/Hydrogen and Cracked Ammonia Combustion

**DOI:** 10.1021/acs.energyfuels.5c02759

**Published:** 2025-09-29

**Authors:** Giovani Battista Ariemma, Giancarlo Sorrentino, Mara de Joannon, Raffaele Ragucci, Pino Sabia

**Affiliations:** Institute of Sciences and Technologies for Sustainable Energy and Mobility (STEMS-CNR), Naples 80125, Italy

## Abstract

Ammonia is a promising energy carrier for energy system decarbonization, although several drawbacks affect its combustion process. Coupling moderate or intense low-oxygen dilution (MILD) combustion with the use of high reactivity fuels allows to improve NH_3_ combustion. In particular, H_2_ addition may be a feasible strategy, considering the high proportion of H_2_ achievable by NH_3_ partial cracking. The present study focuses on MILD combustion effectiveness in ensuring high stability and low-NO_*x*_ emissions for NH_3_/H_2_ blends. Influence of both equivalence ratio and H_2_ addition was experimentally investigated in a cyclonic reactor. Furthermore, the results were directly compared with those obtained with cracked NH_3_ mixtures (NH_3_/H_2_/N_2_). Results for NH_3_/H_2_ blends strengthen the fuel flexibility of the cyclonic reactor, which allows total conversion of the fuel mixtures by ensuring operating temperatures always lower than 1400 K, independently of the equivalence ratio and the fuel blend composition. In particular, H_2_ addition increases NH_3_ reactivity, whereas increasing NO_*x*_ emissions with respect to pure ammonia. Instead, for pure H_2_ and pure NH_3_, they always stay lower than 40 and 100 ppm, respectively. For cracked NH_3_ mixtures, the fuel dilution content by N_2_ does not affect the NH_3_/H_2_ combustion behavior under MILD conditions. Instead, for 100% NH_3_ cracking (75%H_2_-25%N_2_ mixture), H_2_ dilution by N_2_ entails a more uniform reaction zone than not diluted H_2_ case, further limiting NO_*x*_ formation by avoiding the occurrence of hot-spot regions within the reactor.

## Graphical abstract

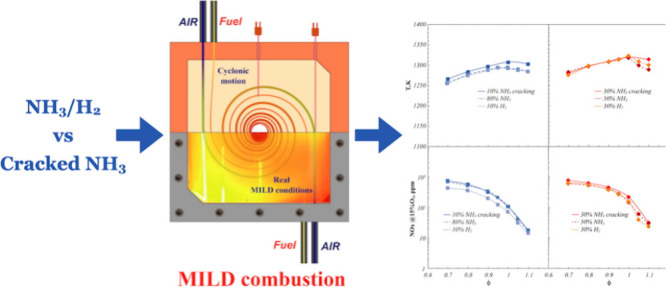

## 1 Introduction

Environmental issues deriving from the use of fossil fuels in power and energy sectors are among the most relevant industrial challenges. In this context, incorporating renewable energy sources is crucial for meeting the increasing energy demand and enhancing sustainability in energy conversion, distribution, and usage.^1^ However, the nonprogrammable and often local nature of renewables poses some essential challenges for matching energy supplies to demands, in both time and space.

A promising and effective solution to guarantee security and flexibility to the power generation based on renewable sources is the use of suitable back-up systems.^2–4^ In this respect, the energy storage in a chemical form, especially through carbon-free energy vectors, represents a key element with respect to the energy system decarbonization, balance, and grid integration.^4–6^ Indeed, chemical energy carriers benefit of relevant storage capabilities, in terms of energy density and discharge time duration.^2,3,7^

Therefore, the growing interest of the scientific community to reach the zero-emission target resulted in increasing concern about the potential role of no-carbon energy vectors. In this respect, hydrogen unquestionably plays a prominent role, as a carbon-free candidate to fuel power and heat generation devices. However, many techno-economic barriers hinder a straightforward transition to a hydrogen economy.^8–10^

An effective alternative is to chemically store hydrogen in the form of ammonia (NH_3_), easily liquifiable at 8 bar and at room temperature, and thus easy to be transported and distributed on a large scale.^11–13^

On the other hand, ammonia suffers several drawbacks, especially with respect to its corrosive nature and, related to its use as fuel, scarce combustion properties.^14,15^ These latter represent a hindrance to its effective use in both stationary plants and transportation systems based on conventional combustion apparatus, since suffering stabilization issues and high NO_*x*_ levels.^14,16^

Specifically, NO_*x*_ emissions are usually limited by different approaches. Among them, exhaust gas after-treatment techniques, such as selective catalytic and noncatalytic reduction,^17^ dual-stage combustion systems,^18^ and reactant humidification techniques,^19^ are broadly employed. Although such strategies are potentially able to ensure effective NO_*x*_ reduction, they are characterized by several limitations, especially in terms of flexibility and technological complexity.

On the other hand, blending NH_3_ with more reactive fuels is the main strategy to increase ammonia combustion stability,^20–23^ especially in traditional combustion systems. Nevertheless, NO_*x*_ emission levels even more represent an open issue, since a boosted NO_*x*_ production characterizes NH_3_ blends with respect to pure NH_3_.^24,25^

In this framework, the use of NH_3_/H_2_ blends represents the most attractive possibility within the perspective of a carbon-free energy production system.^26,27^ In fact, both of them are potential clean fuels, characterized by complete combustion products (N_2_, H_2_O) that are harmless for the environment. H_2_ shows wide flammability limits and high heat value per mass unit, contrary to NH_3_, but suffering storage and transportation issues, differently from NH_3_.

Moreover, since NH_3_ is an effective hydrogen carrier that, in turn, is an excellent combustion promoter for ammonia itself, directly obtainable via NH_3_ partial cracking,^28^ cracked NH_3_/H_2_/N_2_ blends can be seen to be an effective fuel source, able to overcome the intrinsic limitation of its components.

In this respect, in situ producibility of molecular hydrogen through ammonia partial dissociation is a very promising approach.^29^ It allows the production of NH_3_-based fuel mixtures with combustion characteristics tending to conventional fuels, thus needing only marginal modifications of conventional burner design. In this respect, partially dissociating ∼30% of the ammonia to generate an NH_3_/H_2_/N_2_ blend yields combustion stability limits comparable to that of typical hydrocarbon fuels.^19^

Indeed, H_2_ is the most used additive to assist in ammonia combustion in the SI engine. An H_2_ mass fraction of ∼10% is required for the NH_3_/H_2_SI engine to acquire comparable performances with that of a neat gasoline SI engine,^30^ although undesirable elevated unburned NH_3_ and NO_*x*_ emissions remain an issue to be faced with.^31^

Recent literature studies on spark ignition engines fueled with gaseous ammonia blends in a wide range of hydrogen mole fractions and mixture compositions showed improvements in the cyclic stability and in avoiding misfiring. Higher hydrogen fractions result in depleted efficiency, attributed to higher wall heat losses.^32^ In the same context, some studies aimed at improving the knowledge gap related to the understanding of turbulent-flame interaction of ammonia-hydrogen-based fuels, revealing that the flame curvature remains relatively similar between NH_3_ and ammonia/hydrogen blends.^33^

Furthermore, NH_3_/H_2_ blends are also being considered as fuels for application in gas turbine combustion systems.^34^ For these systems, ammonia combustion strategies are mainly based on the partially premixed combustion, capable of reaching stable oxidation with reasonable NO emission levels. However, further efforts are still needed to evaluate other harmful pollutant emissions such as N_2_O and NO_2_. Recently, Pashchenko^35^ reviewed the different aspects related to the utilization of ammonia as a fuel for gas turbines by including prospective technology for ammonia usage through the thermochemical transformation into hydrogen-rich gas. Yao et al.^36^ highlighted the key role of ammonia-hydrogen blends as carbon-free energy carriers for GTs, underlying the strong dependence of the combustion performance on the blending ratio, with an important role in several configurations of wall cooling effects. In this respect, the possible integration of the combustion unit with a chemical recuperator seems to be a promising option for dual-fuel systems. In this context, several configurations were used to mimic gas turbine combustors with ammonia/hydrogen blends, including circular gas turbine combustion burners,^37^ Rich–Quench–Lean with humidified ammonia/hydrogen^38^ and porous media burners.^39^

Despite that, fuel mixtures derived from NH_3_ partial cracking also contain, along with NH_3_ and H_2_, molecular nitrogen (N_2_), which imposes the use of diluted fuel blends. Such an aspect can potentially hinder their direct utilization in conventional combustion systems relying on traditional flame stabilization mechanisms, especially in terms of process stabilization.^40^

In fact, Shohdy et al.^41^ highlighted the key role of ammonia cracking grade in enhancing the stability limits and NO*_x_* emissions with respect to pure ammonia in a swirl-stabilized premixed flame. In this respect, although ammonia cracking is beneficial to enhance the lean blow-off limit and the overall burning velocity, its impact on pollutant emissions and flame stability is detrimental for a cracking grade lower than 20%, making these flames more prone to thermoacoustic issues than pure ammonia flames. Mei et al.^42^ showed that both partial ammonia cracking strategy and operating pressure increase can effectively enhance ammonia combustion, although cellular instabilities and a dramatic nonmonotonic NO increase occur as the cracking ratio and pressure increase. Furthermore, Khateeb et al.^43^ showed that nitrogen dilution due the partial ammonia cracking slightly increases the equivalence ratios at flashback, lean and rich blowout for premixed swirl burner, with a tangible but not severe effect on the combustion process stability limits.

In this respect, traditional combustion systems still need huge efforts with respect to the utilization of ammonia, its blends, and fuel mixtures derived from ammonia partial cracking to ensure stable and efficient thermochemical conversion, with acceptable NO_*x*_ emissions and unburned NH_3_.

These requirements may be effectively ensured through innovative combustion technologies, i.e., MILD combustion,^44^ based on the oxidation process mainly controlled by local mixture ignition mechanisms under moderate temperature^44^ instead of deflagrative/diffusive structures. Such a characteristic makes MILD combustion intrinsically flexible, enabling the possibility of using any fuel of interest with excellent performances in a wide range of operative conditions. It has been already proven its effectiveness with reference to gaseous^45^ and liquid hydrocarbons,^46^ biogas,^47^ alcohols,^48^ and ammonia.^49^ With respect to this latter, recent studies already reported excellent performances in terms of NO_*x*_ levels and process stability for both pure ammonia and its blends with more reactive fuels.^49,50^ However, despite the large number of works reported in the literature about the use of H_2_-based fuel mixtures under MILD conditions,^50–55^ the use of NH_3_/H_2_ blends and even more of fuel mixtures derived from NH_3_ partial cracking still needs thorough investigation.

In this framework, this study aims to provide relevant deep insights about process stability and emissions of NH_3_/H_2_ blends, ranging from pure NH_3_ to pure H_2_, under highly locally diluted and preheated conditions. In this respect, this study comprehensively investigates the combination of NH_3_/H_2_ blends with MILD combustion technology. This combination is significant due to the potential of NH_3_/H_2_ blends as a carbon-free fuel source and the inherent advantages of MILD combustion in terms of fuel flexibility and reduced emissions.

Furthermore, the influence of the presence of N_2_ in the fuel mixture on NH_3_/H_2_ MILD combustion was investigated, thus highlighting the effectiveness of this technology also with respect to fuel blends directly obtained from NH_3_ partial cracking. In this respect, this work uniquely examines the influence of N_2_ dilution in the NH_3_/H_2_ mixture on the MILD combustion process. The findings provide valuable insights into the feasibility and performance of using partially cracked NH_3_ as a fuel in MILD combustion systems.

Experimental campaigns were carried out in a cyclonic flow reactor under MILD combustion conditions. Operating temperatures, emissions levels (NO_*x*_, NH_3_, and H_2_), and process stability limits were systematically monitored by conducting a systematic investigation of different operating parameters, including the global inlet equivalence ratio (ϕ), NH_3_/H_2_ ratio, and NH_3_ cracking grade. This comprehensive approach allows for a detailed understanding of the interplay between these parameters and their combined effect on system performance.

To highlight the influence of multiple operating parameters, i.e., inlet equivalence ratio, NH_3_/H_2_ ratio, and NH_3_ cracking grade, on the system performance, obtained results allowed identification of the optimal operative conditions to ensure oxidation stability and minimal pollutants for NH_3_/H_2_ mixtures, providing crucial information for the effective design and implementation of MILD burners aimed at efficiently and safely utilizing NH_3_/H_2_ blends or cracked ammonia.

## 2 Experimental Setup and Methodology

Experimental tests were carried out in the Laboratory Unit CYclonic (LUCY) burner, extensively described in previous works.^47,56^ Briefly, it is a fuel flexible prismatic chamber with a volume of 2000 cm^3^, operating under MILD combustion conditions. These are achieved inside the reactor by continuously preheating and diluting the incoming fresh reactants by a tailored cyclonic flow field, which realizes an effective internal recirculation of combustion products. Specifically, to ensure the cyclonic flow field establishment, the reactant mixture is fed to the burner by means of two couples of tubes and placed in antisymmetrical position on two opposite walls of the combustion chamber (Figure 1). Such a feeding configuration, along with the exit of flue gases, orthogonally placed with respect to the inlet reactants (Figure 1), induces and stabilizes a toroidal motion field.

**Figure 1:**
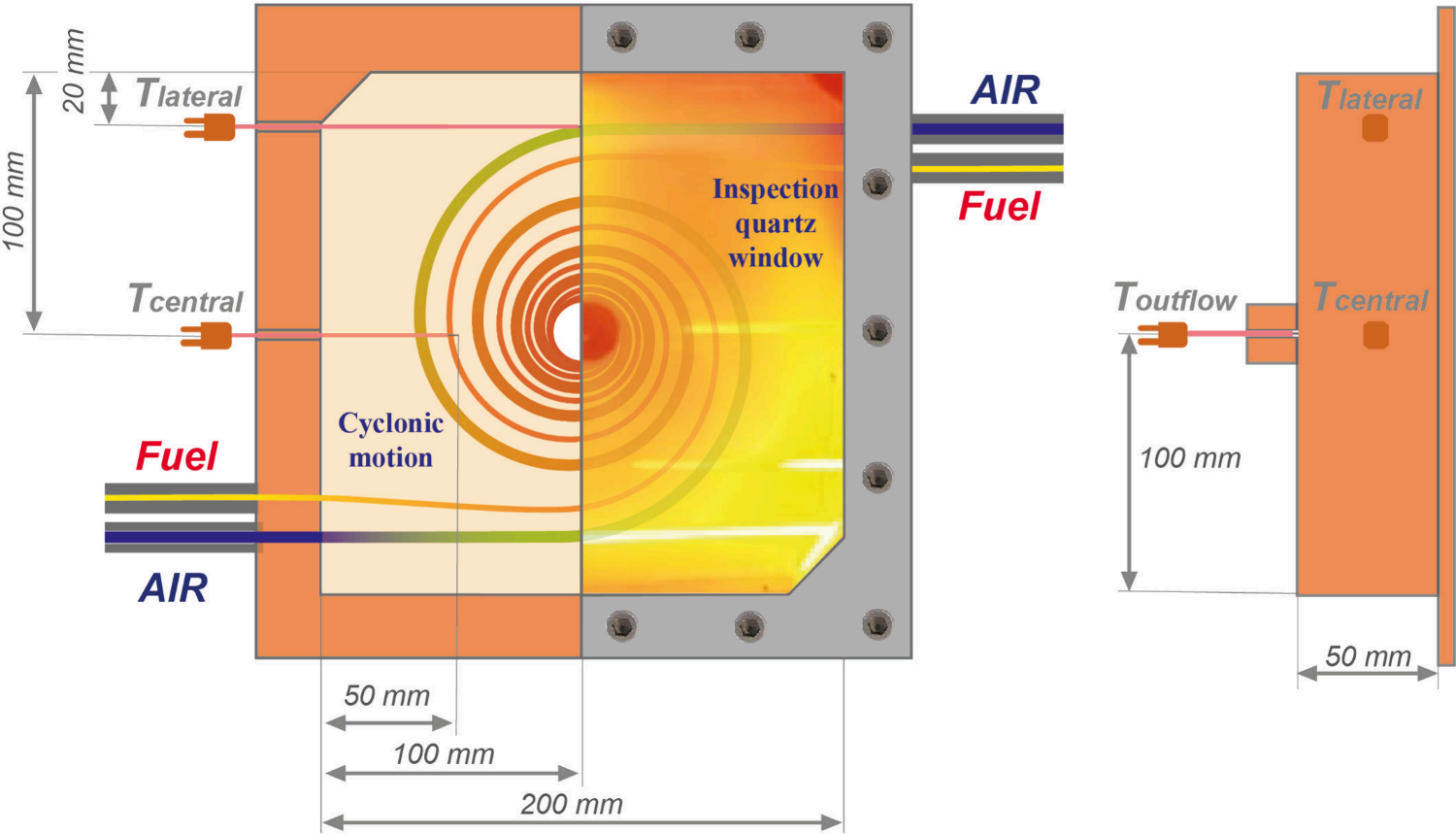
Midplane section of the burner.

The cyclonic burner employed in this study represents a scale-bridging configuration that exhibits quasi-industrial characteristics while enabling precise control over the boundary and operating conditions. This setup closely resembles the targeted industrial applications (such as boilers, gas turbines, and furnaces) but avoids the complex interactions typical of fully industrial systems. Such characteristics are essential for transferring information from laboratory test cases to practical applications and for facilitating the validation of physical submodels.

The reactive flow temperatures were monitored by means of two N-type thermocouples (*T*_lateral_ and *T*_central_), located at the midplane of the chamber, and by a third N-type thermocouple (*T*_outflow_), located at the center of the outflow section of the reactor to detect the characteristic temperature of the flue gases. The nominal accuracy of each thermocouple is assumed to be 0.75% of the measured temperature value. For each test point, operating temperatures were collected at an acquisition rate of about 1 kHz. A minimum of 500 values was acquired and averaged, thus resulting in a maximum uncertainty below 1 K for the whole measured temperature range.

As a result of the uniform temperature distribution and the reduced temperature gradients within the combustion chamber, which essentially entails flat radial and transversal temperature profiles in the reactor and equal to the flue gases one,^57^ the temperature measured at the outflow section is here reported as a reference parameter to compare experimental outcomes.

The experimental campaign was carried out to investigate the suitability of MILD combustion as a reliable technology enabling the use of NH_3_, H_2_, and their blends as innovative energy vectors. Specifically, starting from pure NH_3_, the effects of progressively adding H_2_ to NH_3_/air mixtures were highlighted, directly comparing the experimental evidence with those obtained for NH_3_/H_2_/N_2_ fuel mixtures derived from NH_3_ cracking.

Process stability, operating temperatures levels, and emissions (NO_*x*_, NH_3_ slip, and H_2_) were analyzed, as a function of the global inlet equivalence ratio (ϕ), H_2_ volumetric percentage in the fuel mixture (%H_2_), and simulated NH_3_ cracking ratio (%cracking).

In particular, the global equivalence ratio (ϕ) was defined based on the reactions 4NH_3_ + 3O_2_ → 2N_2_ + 6H_2_O and 2H_2_ + O_2_ → 2H_2_O, in agreement with eq 1:

$$\phi =\frac{\left(\mathrm{fuel}/O_{2}\right)}{(\mathrm{fuel}/O_{2}{)}_{\mathrm{stoich}.}}=\frac{3}{4}({\mathrm{NH}}_{3}/O_{2})+\frac{1}{2}(H_{2}/O_{2})$$ 1

with NH_3_, H_2_, and O_2_ inlet volumetric flow rate fed to the cyclonic burner.

The %H_2_ in the fuel mixture was varied from pure NH_3_ to pure H_2_, by gradually substituting NH_3_ with H_2_ in the fuel stream, while the %cracking was varied from 10% up to 100%, by keeping constant the nominal thermal power at 7 kW for all the investigated conditions. Both oxidizer and fuel flows were fed to the combustion chamber at ambient conditions (*T*_in_ = 300 K, *p* = 1 atm) by using dedicated EL-FLOW select mass flow controllers supplied by BronkHorst High Tech (nominal accuracy of ± 0.5% Reading Rd plus ± 0.1% Full-Scale FS). In this respect, the investigated experimental conditions are summarized in Table 1.

**Table 1:** Experimental Operative Conditions for the Cyclonic Flow Reactor

parameter	range
fuel mixture (NH_3_/H_2_)	from pure NH_3_to pure H_2_
NH_3_ cracking (%cracking)	10–30–50–100%
equivalence ratio (ϕ)	0.4–1.2
nominal thermal power (P)	7 kW
inlet temperature (*T*_in_)	300 K
operating pressure (*p*)	1 atm
nominal residence time (τ)	0.2–0.6 s

The oxidation process was characterized by sampling the exhaust gases in the chamber outflow section. First, the sampled stream was kept at 400 K to prevent water condensation and NH_3_ solubilization and then fed to a FTIR analyzer (SICK GME700). This allows for NH_3_ slip quantification with a maximum error equal to 4% of the detected value by the principle of TDLS (tunable diode laser spectroscopy) and by using specific light absorption. Afterward, the sampled gas was dried through a water condensation trap and fed to an offline Agilent 490 Micro GC - quad w/four channels analyzer and an online TESTO 350. Specifically, the Agilent micro-GC 490 analyzer was used to measure H_2_, O_2_, and N_2_ volumetric concentrations (on a dry basis) by using a thermal conductivity detector (TCD). It was equipped with four specific capillary columns, including a column Molsieve 5A to separate H_2_, N_2_, and O_2_ species with maximum relative errors around ± 3%. On the other hand, the online gas analyzer TESTO 350 was used to monitor the NO*_x_* emissions (on dry basis), with an estimated error equal to ± 2 ppm in a range of 0–99.9 ppm and ± 5% in a range of 100–2000 ppm.

All the concentrations of detected species were then normalized to 15% vol O_2_. To verify the accuracy and repeatability of experimental measurements, these were replicated three times in different days. Specifically, after stationary conditions were reached for each investigated case, three repetitions of every chemical sampling and analysis procedure were carried out. Such approach results in a maximum error of 5% for the species concentrations. Due to the very low measurements relative errors ensured by the adopted approach, the related error bars for each temperature and species data are included in the width of the related markers in the showed figures.

To prevent the formation of explosive stagnation zones due to possible hydrogen leakages, the cyclonic flow burner was upgraded with a forced ventilation system with respect to the previous setup.^47,49^ The resulting lower boundary temperature around the reactor and the higher heat transfer entailed operating temperatures for pure NH_3_ that were lower than those reported in previous works for the same conditions and without the ventilation system. Nevertheless, this aspect does not affect the generality of the obtained results.

## 3 Experimental Results for NH_3_/H_2_ Fuel Blends

The experimental results were reported in the following section in terms of operating temperatures (*T*), emission levels (NO_*x*_), speciation (NH_3_, H_2_, and O_2_), and process stability, analyzing the influence of the global equivalence ratio (ϕ) and the volumetric H_2_ content (%H_2_) of the reactant mixture. For all the explored cases, fuel mixture composition and resulting NH_3_/H_2_ ratio are summarized in Table 2, while further detailed information about the experimental conditions (inlet flow rates, inlet velocities, residence times, and reactant composition) is reported in Sections 1 and 2 of the Supporting Information.

**Table 2:** NH_3_/H_2_ Fuel Mixture Composition (%v/v) and NH_3_/H_2_ Characteristic Ratios as a Function of the Volumetric %H_2_ in the Fuel Mixture

fuel mixture	%NH_3_	%H_2_	NH_3_/H_2_ ratio
ammonia	100	/	/
5% H_2_	95	5	19
10%H_2_	90	10	9
20%H_2_	80	20	4
30%H_2_	70	30	∼2.33
40%H_2_	60	40	1.5
50%H_2_	50	50	1
60%H_2_	40	60	∼0.67
70%H_2_	30	70	∼0.43
80%H_2_	20	80	0.25
90%H_2_	10	90	∼0.11
95%H_2_	5	95	∼0.05
hydrogen	/	100	0

### 3.1 Influence of the Global Equivalence Ratio (ϕ)

This section analyzes and discusses the influence of the equivalence ratio (ϕ), defined in agreement with eq 1, on the characteristic levels and distribution of operating temperatures (*T*) and stable species (NO_*x*_, NH_3_, H_2_, and O_2_) for all the considered NH_3_/H_2_ fuel mixtures listed in Table 2. In this respect, in Figure 2, operating temperatures for pure NH_3_, pure H_2_, and NH_3_/H_2_ blends as a function of ϕ are reported, with the investigated volumetric fraction of hydrogen (%H_2_) in the fuel mixture varied from 5% up to 100%. Extinction conditions detected for fuel-lean cases are also shown by dashed lines.

**Figure 2:**
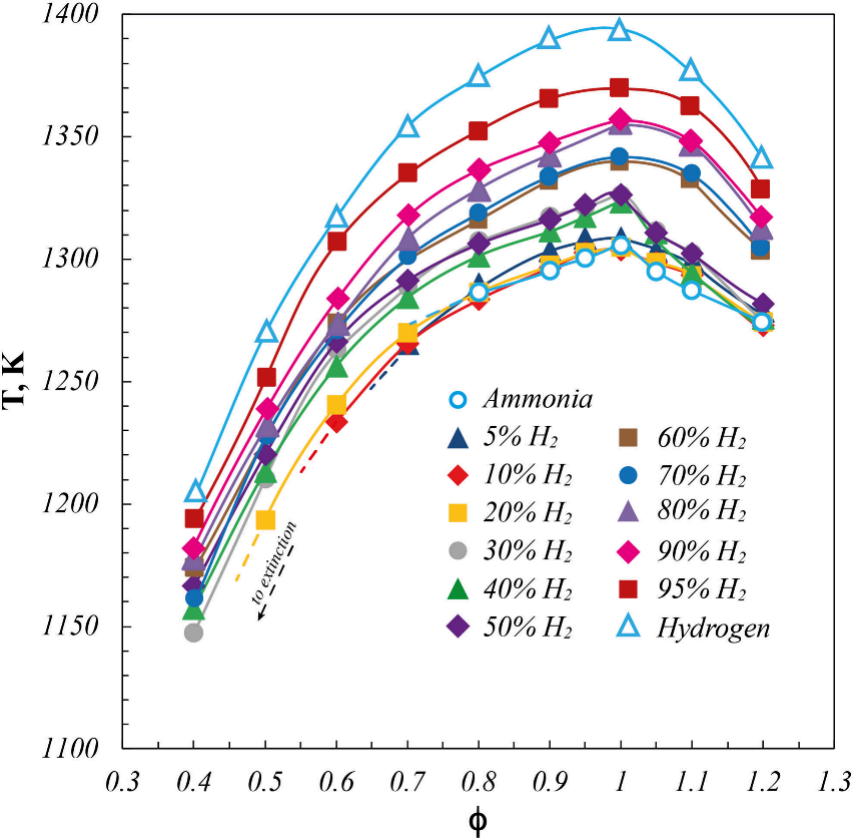
Operating temperatures (*T*) for pure NH_3_, pure H_2_, and NH_3_/H_2_ blends as a function of ϕ. *P* = 7 kW.

For all the reported cases, *T* profiles show a nonmonotonic trend as a function of ϕ, with the maximum located at the stoichiometric condition (ϕ = 1) and decreasing trends toward both fuel-lean and fuel-rich conditions. For pure NH_3_, *T* increases from 1290 K at ϕ = 0.8 to a maximum value of 1310 K at the stoichiometric condition. Afterward, *T* decreases to 1270 K at ϕ = 1.2. Similarly, for pure H_2_, *T* increases from 1210 K at ϕ = 0.4 to a maximum value of 1395 K at ϕ = 1 and then decreases down to 1340 K at ϕ = 1.2. NH_3_/H_2_ blends show similar temperature profiles to the pure fuel cases, with temperature levels between the reference pure fuels. Specifically, temperature levels for NH_3_/H_2_ fuel mixtures follow the increasing hydrogen content (%H_2_) in the fuel mixture, due to the highest adiabatic flame temperature of H_2_ with respect to NH_3_. In this respect, operating temperatures show a nonlinear increase as a function of the %H_2_ in the fuel mixture. Indeed, starting from the pure NH_3_ case up to 50%H_2_, the maximum *T* increase keeps lower than 20 K, whereas a more marked *T* increase is detected by further increasing the %H_2_ in the fuel mixture. This reaches almost 75 and 100 K for 95%H_2_ and 100% cases, respectively, with respect to the pure NH_3_ case.

In this regard, results highlighted almost unchanging operating temperature levels with respect to the fuel mixture composition, with all the investigated cases included in a temperature range of 100 K. This marked fuel flexibility is guaranteed by the peculiar fluid dynamics of the cyclonic burner, which ensures the continuous mixing and dilution of the reactive mixture by the recirculation of combustion products induced by the cyclonic flow field. This behavior entails temperature differences are strongly flattened, as detected by moving from the pure NH_3_ to pure H_2_ case in the whole range of investigated ϕ.

Furthermore, results also highlight the effectiveness of H_2_ as an ammonia fuel enhancer in widening the operating stable window in which the oxidation process stabilization is achieved, with respect to both ϕ and minimum operating temperature. Indeed, within the investigated ϕ range, sudden extinction behaviors occur for pure NH_3_ and NH_3_/H_2_ blends up to %H_2_ = 20 for fuel-lean conditions, as highlighted by dashed lines in Figure 2. For pure NH_3_, extinction phenomena occur at ϕ < 0.8 and *T* < 1290 K (a higher ϕ and lower *T* with respect to previous results obtained for pure ammonia,^49^ as results of the implemented ventilation system for the experimental apparatus, as explained in Section 2), while the H_2_ presence in the fuel blend extends the process stability toward fuel-leaner and lower-temperature conditions. In this respect, extinction phenomena are driven by the recirculated sensible enthalpy and needed chemical times required to promote the autoignition of the reactive mixture, essential to continuously sustain the oxidation process. In particular, since the fresh reactants reach the ignition conditions by mixing with the recirculating exhaust gases, the lower their characteristic temperature, the less effective the preheating of the reacting mixture up to the autoignition conditions. In fact, approaching the fuel-lean extinction limits and operating temperatures decrease, entailing longer chemical times that became comparable with the mixture residence ones. This behavior is confirmed by the evaluation of ignition delay times and minimum operating temperature allowing the reactant mixture ignition of fuel blends for which extinction phenomena were experimentally detected (i.e., pure NH_3_ case and NH_3_/H_2_ blends up to 20%H_2_), reported in Section 3 of the Supporting Information. In particular, when the system approaches extinction conditions, the temperature detected in the region where first reactive kernels form and develop within the reactor (*T*_lateral_ in Figure 1) approaches the minimum temperature required for the mixture ignition. As a result, this condition causes the combustion process stabilization to become very sensitive to the competition between the characteristic chemical and residence times, thus defining a condition in which the process is prone to extinguish.

With respect to the speciation, in Figure 3, the main species detected in the exhausts, normalized at 15% O_2_, for pure NH_3_, pure H_2_, and NH_3_/H_2_ blends are reported as a function of ϕ.

**Figure 3:**
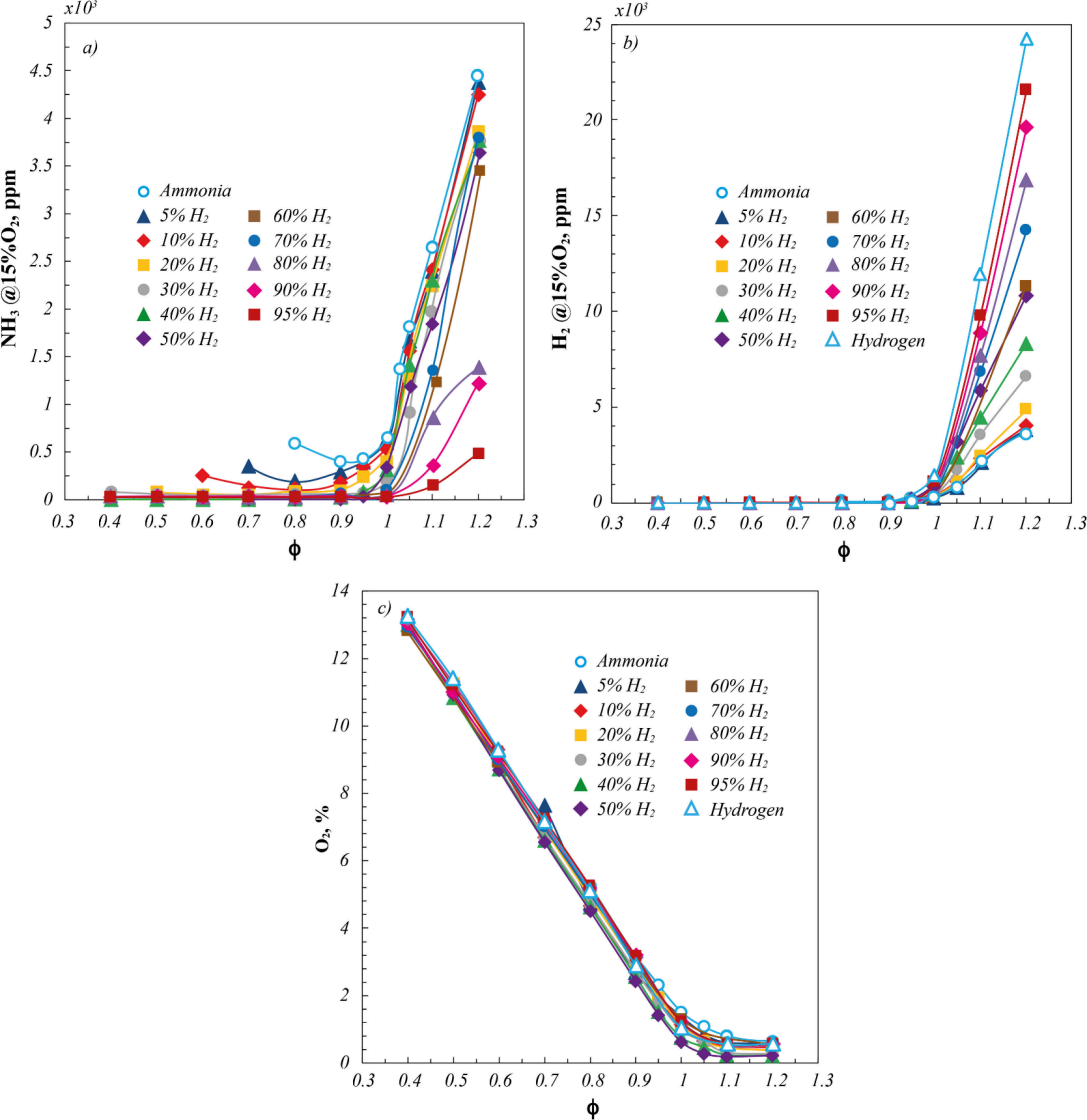
(a) NH_3_, (b) H_2_, and (c) O_2_ emissions for pure ammonia and pure hydrogen (open symbols) and ammonia/hydrogen blends (closed symbols). *P* = 7 kW.

**Figure 4:**
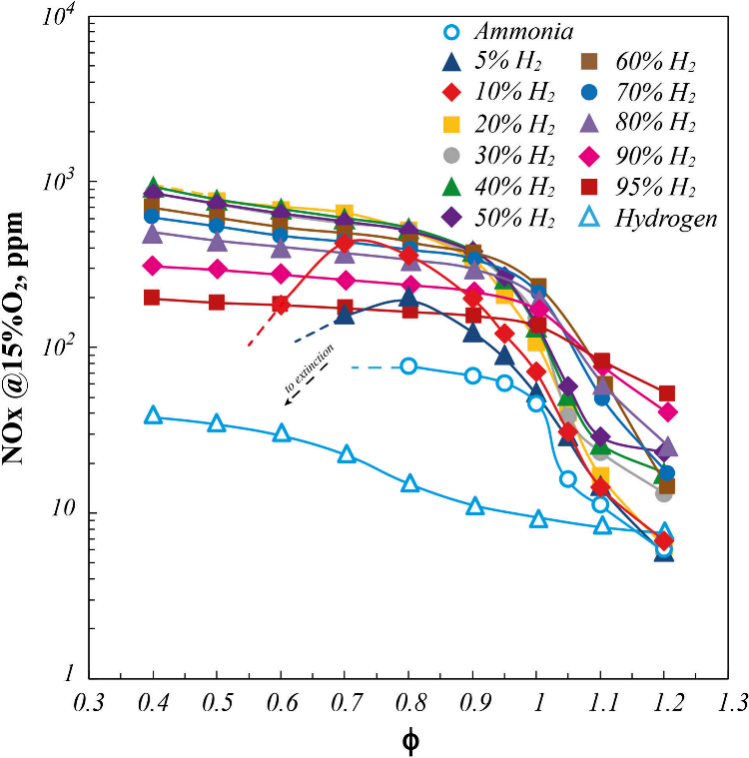
NO_*x*_ emissions for pure NH_3_, pure H_2_, and NH_3_/H_2_ blends as a function of ϕ. *P* = 7 kW.

NH_3_ emissions (Figure 3a) keep very low in fuel-lean conditions, up to about ϕ = 0.9, testifying to the complete NH_3_ conversion for all the investigated cases. Instead, when moving toward fuel-rich conditions, the ammonia slip rapidly increases by approximately 1 order of magnitude. Specifically, a nonmonotonic trend is detected as a function of ϕ for NH_3_ emissions of pure ammonia and NH_3_/H_2_ blends up to about 20%H_2_. In fact, a minimum is detected at ϕ = 0.9 for the pure ammonia case, ϕ=0.8 for blends at 5%H_2_ and 10%H_2_, and at about ϕ = 0.8 for 5%H_2_ fuel mixture, with increasing NH_3_ emissions for both fuel-leaner and fuel-richer conditions. In this respect, the increase of NH_3_ emissions for fuel-leaner conditions is due to the approaching incipient extinction conditions, which entails the incomplete fuel oxidation because of the low reactor temperature and mixture residence times, as previously explained. In fact, in agreement to Figures 2 and 5, these conditions are detected at ϕ equal to 0.7, 0.6, 0.55, and 0.45 for mixture at %H_2_ of 0, 5, 10, and 20, thus explaining the detected NH_3_ slip increase toward ultralean conditions. On the other hand, increasing %H_2_ in the fuel mixture, NH_3_ emissions decrease, especially toward fuel-rich conditions. This behavior is due to both the decreasing NH_3_ content of the inlet reactant mixture and the boosted reactivity entailed by increasing %H_2_, becoming even more marked for hydrogen volumetric content higher than 30%. Indeed, for %H_2_ > 30, NH_3_ emissions detected around the stoichiometric condition are 1 order of magnitude lower than the pure ammonia characteristic ones.

**Figure 5:**
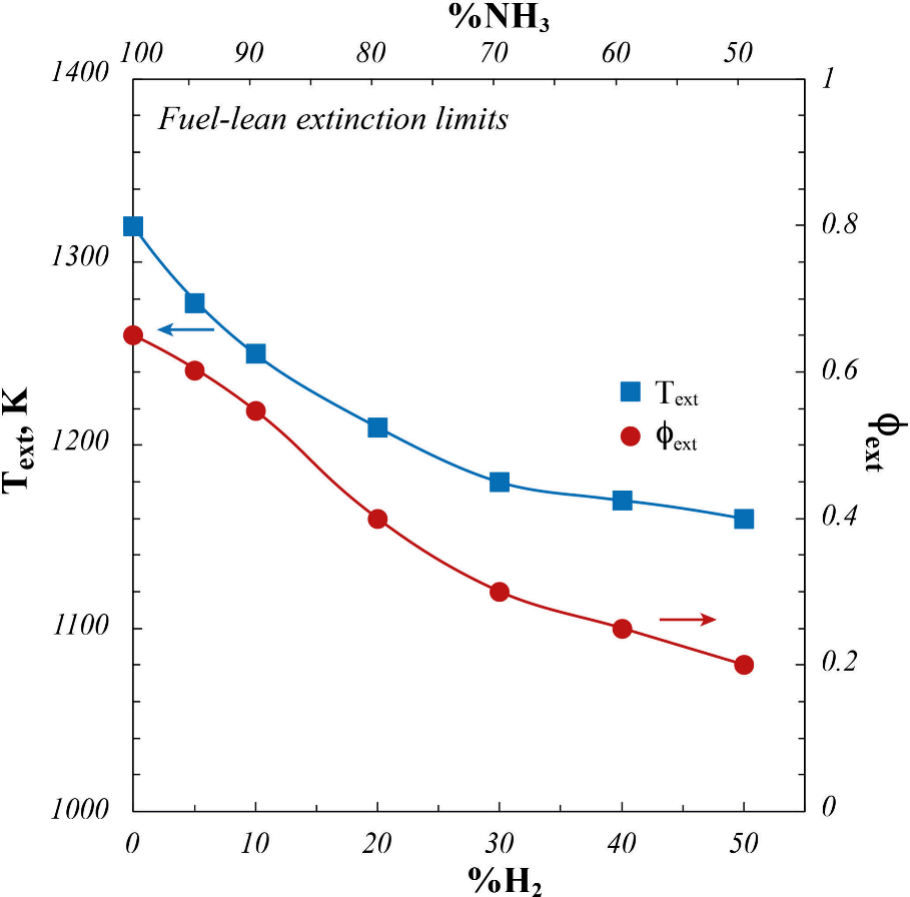
Fuel-lean extinction temperature (*T*_ext_) and equivalence ratio (ϕ_ext_) as a function of %H_2_ and %NH_3_. *P* = 7 kW.

In agreement with the NH_3_ levels is the dependence of H_2_ concentrations in the exhausts with respect to ϕ (Figure 3b), reported on a linear scale. In particular, for ϕ < 0.9, no H_2_ emissions are detected for the cases at %H_2_ > 30, as for NH_3_ emissions due to the marked system reactivity, whereas fuel blends at %H_2_ < 30 and the pure ammonia case are characterized by H_2_ emissions lower than 10 ppm. For ϕ > 0.9, as for NH_3_ emissions, H_2_ emissions linearly increase for all the investigated cases, with levels that follow the %H_2_ in the fuel mixture.

Finally, the O_2_ concentrations (Figure 3c) overlap for all cases, with very slight differences in the fuel-rich side. In particular, the higher the %H_2_ in the fuel mixture the lower the O_2_ concentration, as expected due to the higher reactivity of H_2_ with respect to NH_3_.

On the other hand, in Figure 4, normalized NO_*x*_ emissions are reported in log scale, as a function of ϕ for the investigated cases.

In particular, for pure NH_3_, NO_*x*_ emissions decrease from about 80 ppm at ϕ = 0.8 down to 5 ppm at ϕ = 1.2, while remarkably, NH_3_/H_2_ blends exhibit higher NO_*x*_ emission levels than those obtained with pure ammonia, in agreement with previous findings related to NH_3_/CH_4_ ^58,59^ and NH_3_/alcohol^49^ mixtures. Instead, the lowest NO_*x*_ emissions were detected for pure H_2_, in the whole investigated ϕ range, for which they remain lower than 40 ppm, reaching 10 ppm around the stoichiometric point. In this respect, for the 100%H_2_ case, operating temperatures are always lower than 1400 K, thus strongly differing from the classical behavior of combustion systems operating with hydrogen as fuel under traditional combustion. Indeed, the moderate operating temperatures ensured by the MILD regime also for pure hydrogen allow to strongly limit the thermal NO_*x*_ formation.^60^

Such results consistently report NO_*x*_ levels below 100 ppm for pure ammonia or selected ammonia/H_2_ blends (around the stoichiometric condition), clearly demonstrating the advantages of MILD combustion in terms of NO_*x*_ emissions when compared to conventional swirl-stabilized burners that commonly exceed 200 ppm under optimized conditions.^24,26^

Specifically, the highest NO_*x*_ increase with respect to the pure ammonia case is detected for ammonia blends with 5–10%H_2_, especially under fuel-lean conditions. Moreover, for %H_2_ > 10 and up to 50%H_2_, NO_*x*_ profiles almost overlap, whereas they keep the same trend and lower NO_*x*_ levels for higher %H_2_. As reported in Section 4 of the Supporting Information, reaction rate-based analyses show that the NO_*x*_ formation totally relies on the NH_3_ oxidation that follows the pathways NH_3_→NH_2_→H_2_NO→HNO→NO→N_2_ or NH_3_→NH_2_→NH→HNO→NO→N_2_ depending on the %H_2_ in the fuel mixture. On the other hand, the H_2_ fast oxidation chemistry directly affects the radical pool concentration of the system, specifically boosting H, OH, and HO_2_ radicals’ production. In particular, the marked OH production resulting from the branching mechanism controlled by the H_2_ chemistry (through H+O_2_=OH+O and H_2_O+O=2OH reactions) pushes the NH_2_ conversion toward NH, HNO, and finally NO, by consuming the main species able to act as DeNO*_x_* agent (i.e., NH_2_) and thus resulting in higher NO_*x*_ levels for ammonia blends with respect to pure ammonia.^58^ In particular, due to the pronounced radical production by H_2_, a sudden NO_*x*_ emissions increase characterizes the system even at very low %H_2_ in the fuel mixture.

With respect to the NO_*x*_ profiles, for pure ammonia and blends with %H_2_ > 20, monotonic decreasing trends are detected. Instead, ammonia blends at 5%H_2_, 10%H_2_, and 20%H_2_ show nonmonotonic trends as a function of ϕ, in agreement with NH_3_ emissions profiles for the same cases. In fact, for the fuel mixture with 5%H_2_ NO*_x_* emissions increase from ϕ = 0.6 up to ϕ = 0.8, where the maximum is located, and then decrease for ϕ > 0.8. The 10%H_2_ blend shows an increasing profile moving from ϕ = 0.5 up to ϕ = 0.7, while monotonically decreasing for ϕ > 0.7. Finally, by increasing the %H_2_ in the fuel blend up to 20% NO*_x_* increase from ϕ = 0.4 up to ϕ = 0.6 and decrease for ϕ > 0.7. As previously discussed, for %H_2_ < 30, operating temperatures drastically decrease toward ultralean conditions (ϕ = 0.6 for 5%H_2_, ϕ = 0.55 for 10%H_2_, and ϕ = 0.45 for 20%H_2_), thus becoming comparable to the minimum required levels allowing the fuel mixture ignition in the reference residence time range (Section 3 of the Supporting Information). Under these conditions, the combustion process stabilization becomes very sensitive to the competition between the characteristic chemical and residence times, entailing incomplete fuel conversion and, thus, a decrease in the NO_*x*_ production decrease. This explanation is supported by the NO_*x*_ behavior of mixtures at %H_2_ > 20 that show NO_*x*_ monotonic decreasing trends since not achieving extinction conditions in the investigated ϕ range.

The sensitivity of NO*_x_* emissions with respect to ϕ also shows marked differences in dependence on the considered %H_2_. In this respect, in the fuel-lean side, NO*_x_* emissions first increase from the pure NH_3_ case up to 20–30%H_2_ fuel mixtures, while they decrease for higher %H_2_. Conversely, on the fuel-rich side, the higher the %H_2_ in the fuel mixture, the higher the detected NO*_x_* emissions. Kinetic analyses reported in Section 4 of the Supporting Information highlight that the different NO_*x*_ sensitivity as a function of %NH_3_-%H_2_ and ϕ is to be sought in the different availability of O_2_, OH, NH_2_, and HNO, which directly affect the NO*_x_* production. In particular, in the fuel-lean side, at very low %H_2_ (%H_2_ < 20), the limiting step for the NO*_x_* production is the availability of OH radicals sustaining and boosting the NH_3_ oxidation, independently of the %NH_3_ in the fuel mixture, that always ensures the NH_2_ and HNO radicals’ production. This behavior entails higher NO*_x_* levels for fuel mixtures with progressively increasing %H_2_ up to 20%. By further increasing the %H_2_ above 20%, the decreasing %NH_3_ plays the major role in limiting the availability of NH_2_ and HNO radicals. Therefore, lower NO_*x*_ emission profiles characterize fuel blends at higher %H_2_ and lower %NH_3_. Conversely, in the fuel-rich side, the bottleneck for the NO*_x_* formation is only the OH radicals’ availability, since the deficient O_2_ concentrations. Under these conditions, the higher the %H_2_, the higher the OH radicals’ production and the NH_2_-HNO conversion to NO. This entails higher NO*_x_* emissions for fuel mixtures at higher %H_2_ in fuel-rich conditions. Such considerations are also supported by NO_*x*_ profiles vs %H_2_ shown in Figure 6, which highlight the maximum NO_*x*_ concentration decreases and shifts toward higher %H_2_ in the fuel blend by increasing ϕ.

The qualitative NO_*x*_ trends observed in Figure 4 are also expected in the range of thermal input between 3 and 15 kW. In fact, as already pointed out in previous literature studies on the same lab-scale burner,^61^ the strength of the cyclonic motion and MILD regime stabilization mechanisms is not substantially altered in a range of 3–15 kW.

With respect to N_2_O emissions (not reported in Figure 4 as NO_*x*_), these were preliminarily evaluated for the pure NH_3_ reference case as a function of the equivalence ratio (ϕ). Experimental results showed a monotonic decreasing trend for N_2_O by moving from fuel-lean conditions (ϕ = 0.8) up to slightly fuel-rich ones (ϕ = 1.1). In particular, N_2_O levels (scaled at 15%O_2_) reach the maximum value of about 450 ppm at ϕ = 0.8, then they decrease down to 370 ppm at ϕ = 0.9 and about 100 ppm around the stoichiometric conditions (ϕ = 1). On the other hand, no N_2_O emissions were detected for fuel-rich conditions (ϕ = 1.1). In this respect, preliminary results obtained through CFD analyses (details about the numerical setup and boundary conditions are reported in ref 62) highlighted that the N_2_O formation is mainly localized in the mixing region between the inlet fuel and oxidizer, where the first reactive kernels form and develop. Then, the formed N_2_O is mainly consumed in the recirculation region, where the long residence times ensure the N_2_O decomposition to N_2_.

### 3.2 Influence of %H_2_ in the Fuel Blend

Experimental results analyzed with respect to the equivalence ratio (ϕ) in the previous section clearly highlight the effectiveness of hydrogen in increasing the ammonia reactivity, thus enlarging the stable operating window of the oxidation process. Remarkably, the only nonlinear significant interaction between the two fuels is related to NO_*x*_ emissions production boosted when NH_3_ and H_2_ are blended with respect to the characteristic NO_*x*_ levels of both the pure fuels. With the aim at better emphasizing and analyzing such peculiar behaviors, oxidation process extinction conditions (Section 3.2.1) and NO_*x*_ emissions distribution (Section 3.2.2) are discussed in the following sections with respect to the fuel blend composition (%H_2_-%NH_3_ inlet volumetric concentration).

#### 3.2.1 Oxidation Process Extinction Conditions

Key parameters here considered to analyze the enlargement of the region where stable combustion can be observed by progressively increasing the hydrogen content in NH_3_/H_2_ mixtures are the fuel-lean extinction temperatures (*T*_ext_) and the extinction equivalence ratio (ϕ_ext_) and are analyzed and reported in Figure 5 as a function of both %H_2_ and %NH_3_.

Specifically, the extinction temperature (*T*_ext_) is defined as the average value between the characteristic one of flue gases, detected at the reactor exit (*T*_outflow_ in Figure 1), and the temperature of recirculated combustion products, measured in the central recirculating region of the reactor midplane (*T*_central_ in Figure 1).

In particular, extinction conditions were achieved for fuel mixtures up to 50%H_2_, whereas for higher %H_2_, no extinction phenomena occurred for the investigated ϕ range.

Globally, both *T*_ext_ and ϕ_ext_ are characterized by monotonic decreasing trends as a function of the increasing %H_2_ (decreasing %NH_3_), globally testifying to the widening of the stable region in which the oxidation process can be stabilized by partially substituting H_2_ to NH_3_ in the fuel blend. The extinction temperature decreases from 1320 K starting from pure ammonia (0% H_2_) to 1160 K for a fuel blend at 50%H_2_. Specifically, the *T*_ext_ decrease is almost linear up to 30%H_2_ in the fuel mixture, whereas for higher %H_2_, it only slightly decreases.

The decrease of *T*_ext_ toward about 1100 K clearly highlights that the %H_2_ increase in the fuel blend shifts the minimum stabilization temperature toward the classical crossover temperature,^63^ where the fast chemistry controlled by the high-temperature branching reaction (H+O_2_=OH+O) is replaced by slower branching mechanisms. Such a behavior also clearly demonstrates the leading role of H_2_ with respect to the oxidation process stabilization through a more significant and fast radical pool production. Similarly to *T*_ext_, ϕ_ext_ decreases from 0.65 for pure ammonia (0% H_2_) down to 0.2 at 50%H_2_. In this case, ϕ_ext_ almost linearly decreases as a function of %H_2_ over the whole range of investigated fuel blend compositions.

Analyzed results show that hydrogen addition represents an effective strategy to widen the operating conditions range in which ammonia stable combustion can be effectively stabilized under MILD conditions, especially in terms of reactant mixture composition.

Moreover, when explicitly comparing the stability limits of the cyclonic burner against premixed swirl burner stability ranges reported in the literature by Khateeb et al.,^64^ it appears that such combustion systems report lean blow-off limits at equivalence ratios higher than those achievable in MILD combustion, especially for ammonia fuel fraction higher than 0.6. In this sense, the present MILD burner demonstrated significantly wider stability limits (from ϕ = 0.2 to 1.2), also when compared to conventional lean blow-off equivalence ratios of ϕ ≈ 0.6–0.8, underlining the enhanced stability and broader operating window of MILD combustion.

#### 3.2.2 NO_*x*_ Emission Distribution

With respect to NO_*x*_ levels and distribution, Figure 6 shows NO_*x*_ emissions in linear scale as a function of the %H_2_ and %NH_3_ inlet concentration for several fuel-lean conditions (ϕ = 0.7–0.95) and the stoichiometric one (ϕ = 1).

**Figure 6:**
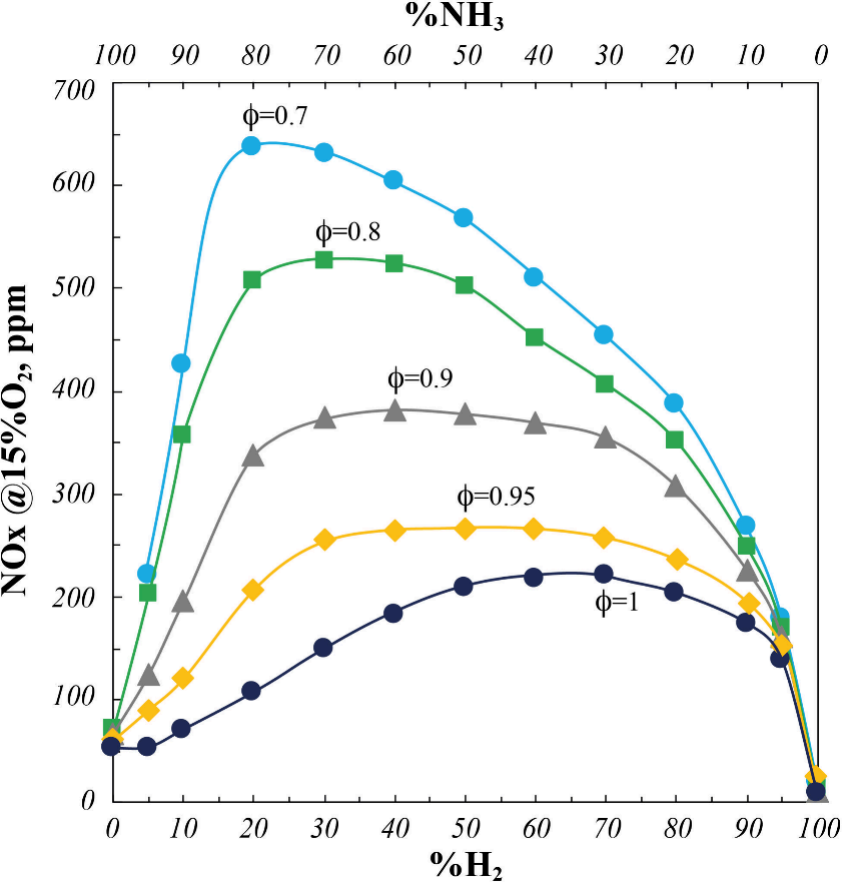
NO_*x*_ emissions as a function of %H_2_-%NH_3_ for 0.7 < ϕ < 1.

First, higher NO_*x*_ emissions characterize NH_3_/H_2_ blends than both the pure ammonia and pure hydrogen, in the whole range of considered blending ratios, with nonmonotonic trends as a function of the blending ratio itself. In particular, for a fixed ϕ, when H_2_ is progressively added to pure ammonia, the NO_*x*_ increases and then decreases, while for a fixed %H_2_, the NO_*x*_ decreases by increasing ϕ, in agreement with data analyzed in Figure 4. Furthermore, the maximum concentration decreases and shifts toward higher %H_2_ in the fuel blend by increasing ϕ, moving from about 650 ppm and 20%H_2_ at ϕ = 0.7 up to 220 ppm and 70%H_2_ at ϕ = 1.

In particular, NO_*x*_ emissions suddenly and almost linearly increase with %H_2_ up to 20%H_2_ only for the fuel-leaner cases, respectively, at ϕ = 0.7–0.8–0.9, whereas they more gradually increase for higher ϕ. Moreover, for ϕ > 0.8 and %H_2_ > 20, NO_*x*_ profiles almost show a plateau that extends up to %H_2_ = 60, in agreement with NO_*x*_ profiles reported in Figure 4.

As explained with respect to Figure 4, the different NO_*x*_ distribution as a function of %NH_3_-%H_2_ and ϕ is due to the different availability of O_2_, OH, NH_2_, and HNO radicals that strongly affect the NO_*x*_ formation and DeNO_*x*_ reactions,^65^ as also highlighted in previous works.^49,59^ In this respect, the main reactions involved in the NO production and consumption (Section 4 of the Supporting Information) highlight the peculiar interplay between the NH_2_ and HNO radicals, deriving from the NH_3_ oxidation pathway (NH_3_→NH_2_→H_2_NO→HNO→NO→N_2_, NH_3_→NH_2_→NH→HNO→NO→N_2_), and the OH radicals, whose formation is supported by the H_2_ chemistry. In particular, the different availability of O_2_, OH, NH_2_, and HNO in dependence on the %H_2_ and ϕ entails nonlinear trends for the main reactions directly involved in the NO_*x*_ formation (HNO+OH=NO+H_2_O, HNO+O_2_=NO+HO_2_, H_2_NO+OH=HNO+H_2_O, NH+OH=HNO+H) and consumption (NH_2_+NO=N_2_+H_2_O, NH_2_+NO=NNH+OH), thus explaining the nonmonotonic NO_*x*_ emissions trend experimentally detected as a function of %H_2_.

In particular, the higher the NH_2_ availability, the higher the NO consumption through DeNO*_x_* reactions (NH_2_+NO=N_2_+H_2_O and NH_2_+NO=NNH+OH). Specifically, for the ϕ = 0.7 case, the large availability of O_2_ and OH radical results in a boosted NH_2_ conversion to NO, also at high %NH_3_. This entails the maximum of NO_*x*_ is located at relatively low %H_2_ (i.e., %H_2_ = 20). On the other hand, for %H_2_ < 20, the decreasing availability of OH radicals sustaining and boosting the NH_3_ conversion to NO entails a decreasing trend for NO_*x*_. Instead, for %H_2_ > 20, the decreasing %NH_3_ plays the major role as a limiting factor for NO_*x*_ production, as previously discussed with reference to Figure 4.

With respect to ϕ, for ϕ > 0.7, both O_2_ and OH radical concentrations decrease, thus resulting in lower NO_*x*_ emissions because of the boosted DeNO*_x_* reactions. Under these conditions, higher %H_2_ are needed to boost the NH_2_ conversion toward NO, entailing the NO_*x*_ maximum shift toward higher %H_2_ by increasing ϕ.

It is noteworthy that the hydrogen addition to pure ammonia results in an even more marked NO_*x*_ increase than that detected due to methane addition and comparable to alcohols one.^49^ Specifically, at ϕ = 0.7, the 10% methane blends resulted in a NO_*x*_ increase with respect to the pure NH_3_ of about 250 ppm, while the addition of the same amount of H_2_ entails a NO_*x*_ increase of about 350 ppm. This behavior confirms the key role of the fuel reactivity on the increase in NO_*x*_ emissions when more reactive fuels are blended with ammonia.

## 4 Cracked NH_3_ Mixtures

Combustion characteristics of NH_3_/H_2_/N_2_ fuel mixtures derived from different NH_3_ cracking grades were investigated and reported in this section. The aim is to highlight the performance of the cyclonic burner with NH_3_/H_2_ fuel blends partially diluted by N_2_, since being a direct product from NH_3_ cracking processes.

Specifically, fuel mixtures derived from 10 to 30–50–100% NH_3_ cracking were synthetically prepared by mixing NH_3_, H_2_, and N_2_, coherently with the volumetric compositions and NH_3_/H_2_ characteristic ratio summarized in Table 3. In particular, the latter is defined as the ratio between the volumetric concentrations of NH_3_ and H_2_ in the cracked fuel mixture.

**Table 3:** NH_3_/H_2_/N_2_ Fuel Mixture Composition (%v/v) and NH_3_/H_2_ Characteristic Ratios as a Function of the NH_3_ Cracking Percentage

%cracking	%NH_3_	%H_2_	%N_2_	NH_3_/H_2_ ratio
10	81.82	13.64	4.54	∼6
30	53.85	34.61	11.54	∼1.56
50	33.33	50.00	16.67	∼0.67
100	0	75.00	25.00	/

In Figure 7, operating temperatures (*T*) and NO_*x*_ emissions for the investigated cracked NH_3_ mixtures are reported as a function of ϕ. Globally, *T* profiles (Figure 7a) agree with those characterizing NH_3_/H_2_ mixtures (Figure 2), with a nonmonotonic trend as a function of ϕ and the maximum located at ϕ = 1.

**Figure 7:**
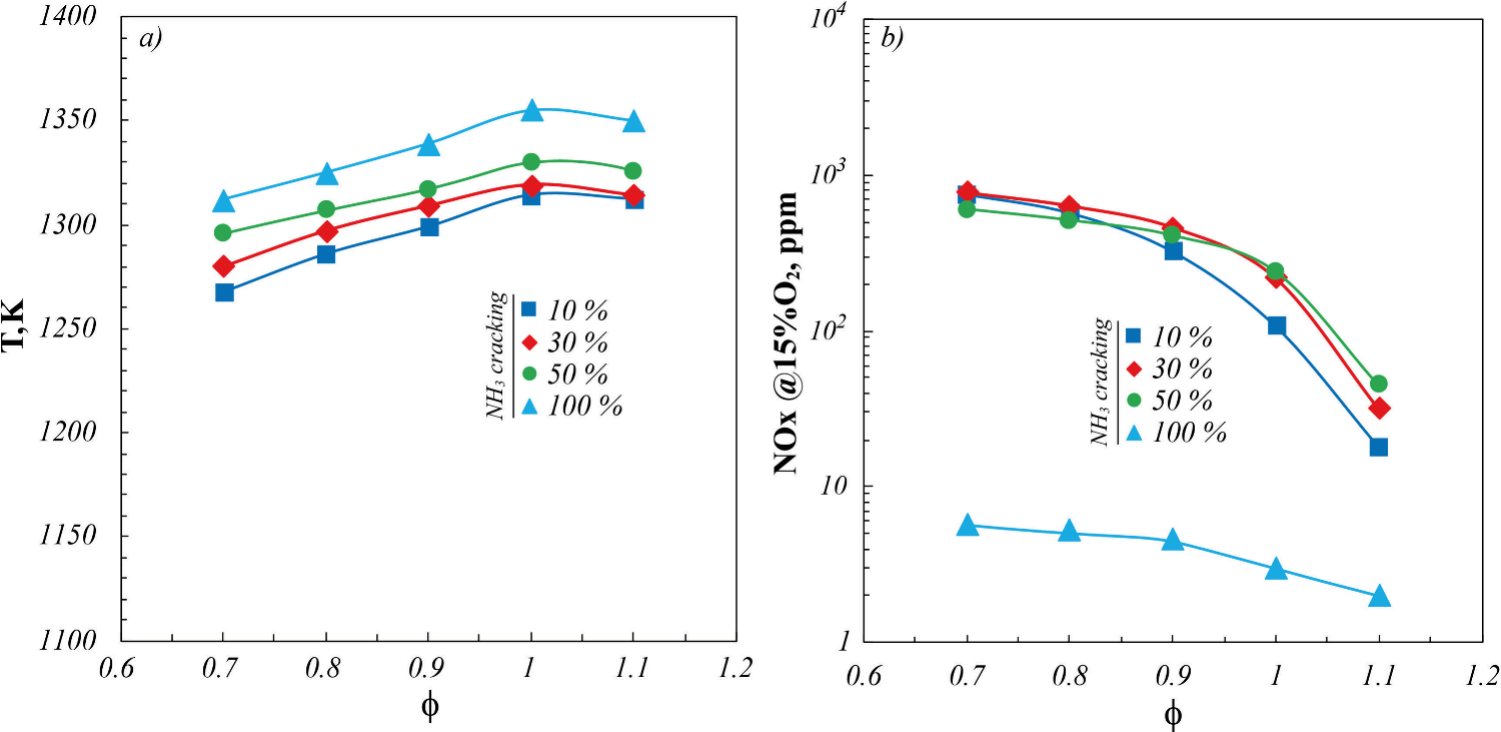
(a) Operating temperatures and (b) NO_*x*_ emissions for fuel mixtures derived from several NH_3_ cracking percentages (%cracking) as a function of ϕ. *P* = 7 kW.

In particular, temperature levels follow the %cracking of the fuel mixture, as a result of the increasing %H_2_ by increasing the cracking percentage (Table 3). In this respect, the wide fuel flexibility of the cyclonic burner makes negligible the effect of simultaneous increase of the fuel dilution (%N_2_), entailing the maximum temperature differences among all of the investigated NH_3_/H_2_/N_2_ fuel mixtures to be lower than 50 K, in the whole range of investigated ϕ.

With respect to the NO_*x*_ emission profiles (Figure 7b), a monotonic decreasing trend is detected as a function of ϕ, in agreement with Figure 4. In particular, the lowest NO_*x*_ levels characterize the fuel mixture derived from 100%NH_3_ cracking (75%H_2_-25%N_2_) in the whole range of investigated ϕ.

On the other hand, fuel mixtures with 10% and 30% NH_3_ cracking show higher NO_*x*_ emissions for 0.7 < ϕ < 0.8, whereas for ϕ > 0.8, higher NO_*x*_ levels are detected for fuel mixtures with 30% and 50% NH_3_ cracking. Such a behavior totally agrees with experimental evidence analyzed and discussed in Section 3.2.2 for NH_3_/H_2_ blends, with the NO_*x*_ emissions maximum shifting toward higher %H_2_ (i.e., higher %NH_3_ cracking) as ϕ increases.

Results reported in Figure 7 show that the inherent fuel dilution of cracked NH_3_ mixtures does not affect the general combustion characteristics of NH_3_/H_2_ blends under MILD conditions. Such evidence can be ascribed to the intrinsic fuel flexibility of MILD combustion and, to a lesser extent, to the moderate dilution content (%N_2_) of cracked NH_3_ mixtures (Table 3).

Moreover, to isolate the effects of fuel composition (i.e., NH_3_/H_2_ ratio and associated cracking) from those due to changes in the inlet dilution, the emissions trends reported in Figures 7b and 11b have been also normalized at the same value of the inlet dilution condition, for each NH_3_ cracking ratio. Such results (reported in Section 5 of the Supporting Information) clearly highlight that the normalized NO_*x*_ trends with respect to ϕ and %cracking overlap with those obtained without normalization, testifying to negligible impact of the different inlet dilution levels of the investigated NH_3_ cracking mixtures. In fact, such comparable trends confirm that the internal dilution effects induced by the cyclonic motion largely mitigate the effect of variations in inlet dilution.

This aspect is better shown in Figure 8, in which operating temperatures and NO_*x*_ emissions detected for NH_3_ cracking mixtures are compared with the ones obtained for NH_3_/H_2_ fuel blends with similar %NH_3_ or %H_2_. Specifically, to provide a fair and meaningful comparison, NH_3_/H_2_ binary mixtures with similar NH_3_ or H_2_ content (as reported in Table 3) of the NH_3_ cracked mixtures considered in Figure 8 were selected. This approach allows us to isolate the effects of cracking (i.e., in situ hydrogen production) from the direct use of NH_3_/H_2_ mixtures under conditions that deliver the same thermal power to the system.

**Figure 8:**
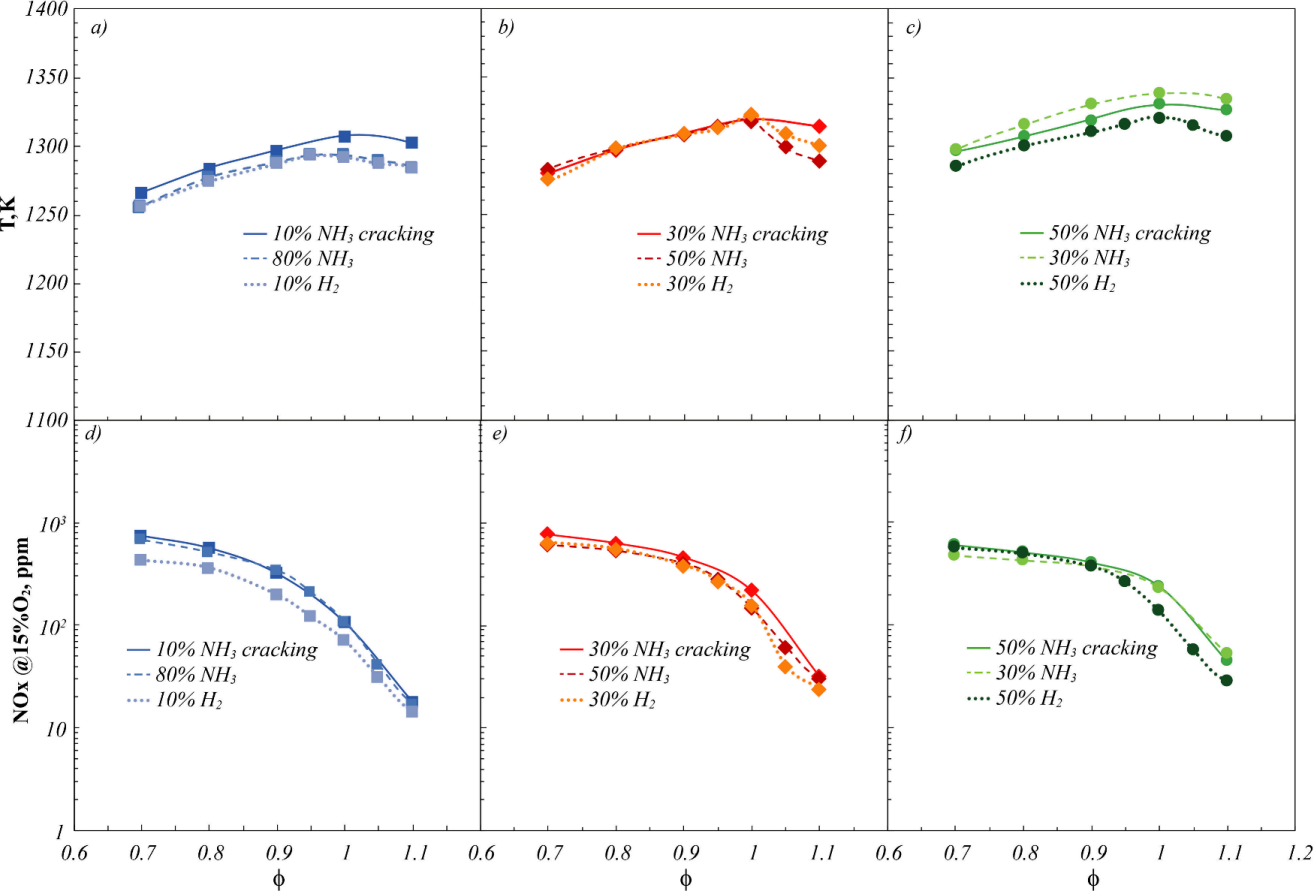
Comparison of (a–c) operative temperatures and (d–f) NO_*x*_ emissions for fuel mixture derived from several NH_3_ cracking percentages and NH_3_/H_2_ blends with similar NH_3_ or H_2_ composition. *P* = 7 kW.

Specifically, operating temperatures (Figure 8a–c) almost overlap for all of the reported cases, with congruent temperature profiles and comparable levels. Indeed, temperature differences among the reported cases always keep lower than 10–15 K. In this respect, the strong internal recirculation of combustion products induced by the cyclonic flow motion flattens the influence of different fuel mixture composition and inlet dilution level.

Similarly, the NO_*x*_ emission profiles (Figure 8d–f) show very slight differences among the compared cases. In particular, with reference to Figure 8d, lower NO_*x*_ emissions characterize NH_3_/H_2_ blends at 10%H_2_ with respect to the 10% NH_3_ cracking mixture, whereas this latter shows overlapping NO_*x*_ levels with the NH_3_/H_2_ blends at 80%NH_3_. Such a difference can be ascribed to the different NH_3_/H_2_ ratio of the reported mixtures, more similar for NH_3_/H_2_ blend with 80%NH_3_ and 10% NH_3_ cracking (Table 3). On the other hand, both NH_3_/H_2_ blends with, respectively, 50%NH_3_ and 30%H_2_ (Figure 8e) show slightly lower NO_*x*_ emission levels than the NH_3_/H_2_/N_2_ mixture derived from 30%NH_3_ cracking. This difference is about 150 ppm, due to both higher %NH_3_ and %H_2_ characterizing this latter with respect to both the reported NH_3_/H_2_ blends. Finally, overlapping NO_*x*_ emissions characterize the 50%NH_3_ cracking mixture with respect to the NH_3_/H_2_ blend with 50%H_2_ up to ϕ = 0.9 (Figure 8f), while for ϕ > 0.9, the cracked mixture shows slightly lower NO_*x*_ levels.

Instead, it is worth noting to compare *T* and NO_*x*_ emissions profiles detected for 100% H_2_ and 100% cracked NH_3_ fuel mixture, reported in Figure 9, since this latter is basically a mixture of pure H_2_ diluted by 25% N_2_.

**Figure 9:**
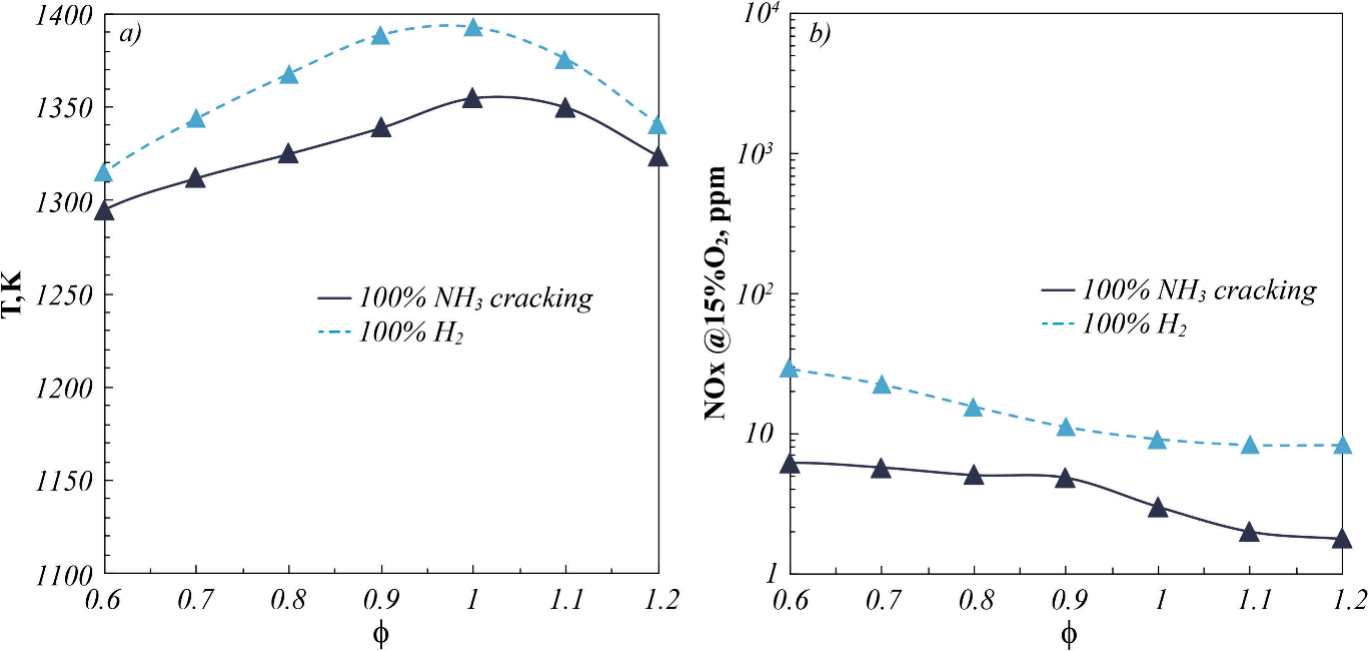
Comparison of (a) operative temperatures and (b) NO_*x*_ emissions for 100%NH_3_ cracking derived mixture and 100%H_2_ as a function of ϕ. *P* = 7 kW.

Specifically, the 100% cracked NH_3_ mixture shows lower temperature levels than the 100% H_2_ case, of about 20–50 K (Figure 9a), and lower NO_*x*_ levels (Figure 9b). The lower operating temperatures may be attributable to the lower adiabatic flame temperature characterizing the fuel mixture derived from 100% NH_3_ cracking, because of the inlet N_2_ dilution. Instead, the lower NO_*x*_ emissions are not simply due to the different total dilution level between 100%H_2_ and 100%NH_3_ cracked cases. Indeed, the H_2_ stream dilution by 25% N_2_ for the 100% cracked NH_3_ fuel mixture entails a more uniform distribution of the reactant mixture within the combustion chamber before reacting. This behavior avoids hot-spot regions within the reactor and entails lower NO_*x*_ formation, as highlighted by CFD analyses reported in Section 6 of the Supporting Information.

To better highlight the different dilution levels characterizing the investigated cases, calculated inlet and local dilution for NH_3_/H_2_ blends and cracked NH_3_ mixtures are shown in Figure 10 as a function of NH_3_/H_2_ ratio, for ϕ = 1 chosen as the reference case. In particular, inlet dilution levels were evaluated with respect to the total inlet N_2_ content (fuel mixture and air), while the local dilution level was determined based on a recirculation ratio *K*_v_ = 5,^66^ a reference value for the reported cyclonic burner.^67,68^

**Figure 10:**
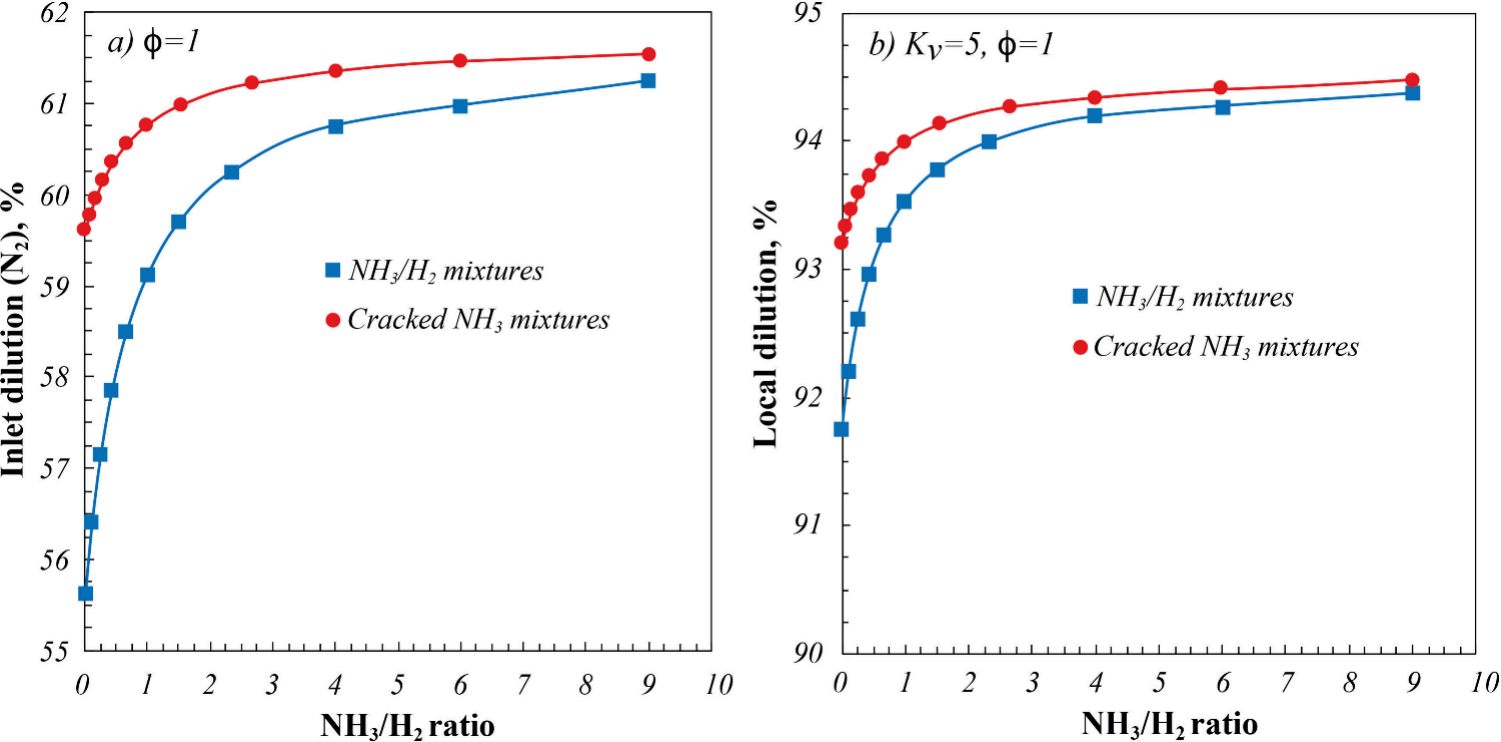
(a) Calculated inlet and (b) local dilution based on a recirculation ratio *K*_v_ = 5 for NH_3_/H_2_ blends and NH_3_/H_2_/N_2_ fuel mixtures derived from NH_3_ cracking as a function of NH_3_/H_2_ ratio. ϕ = 1.

It was evaluated based on the isothermal assumption, which was used to standardize internal dilution effects and facilitate comparison across experimental and modeling studies. Although this assumption may slightly deviate from real reactor conditions due to spatial temperature variations, the moderate and relatively uniform temperature distribution within the reactor and typical of the MILD regime, along with the results obtained by reacting CFD analysis confirm the appropriateness of such a *K*_v_ value.

In particular, higher inlet dilution levels characterize cracked NH_3_ mixtures (Figure 10a), as expected due to the additional N_2_ content of the fuel mixture. On the other hand, the NH_3_/H_2_ ratio increase entails increasing inlet dilution levels, as imposed by the H_2_ and NH_3_ combustion stoichiometry, with decreasing differences between NH_3_/H_2_ mixtures and cracked NH_3_ ones. In particular, inlet dilution moves from about 56%, for pure H_2_ (NH_3_/H_2_ = 0), up to 61% for NH_3_/H_2_ = 9 (equivalent to a fuel mixture with 90%NH_3_), whereas it ranges between 59.5 and 61.5% for cracked NH_3_ mixtures. Coherently, local dilution profiles (Figure 10b) also show the same dependence on NH_3_/H_2_ ratio and lower differences between NH_3_/H_2_ and cracked NH_3_ mixtures, with higher dilution levels as expected due to the considered *K*_v_ = 5.

In this respect, Figure 10 shows that the most pronounced difference of the inlet dilution level characterizes pure H_2_ and 100% NH_3_ cracking cases (NH_3_/H_2_ = 0), thus explaining the detected experimental differences as a function of the NH_3_/H_2_ ratio of the investigated mixtures (Figures 9 and[Fig fig10]). These considerations also apply to different ϕ and *K*_v_ cases. In particular, inlet dilution level differences decrease as ϕ decreases and the level of *K*_v_ increases.

To summarize the systems performance with respect to the use of fuel mixtures derived from cracked NH_3_ mixtures, in Figure 11, operating temperatures and NO_*x*_ emissions are reported for three different ϕ, namely, two fuel-lean conditions (ϕ = 0.8–0.9) and the stoichiometric one (ϕ = 1), as a function of the % NH_3_ cracking.

**Figure 11:**
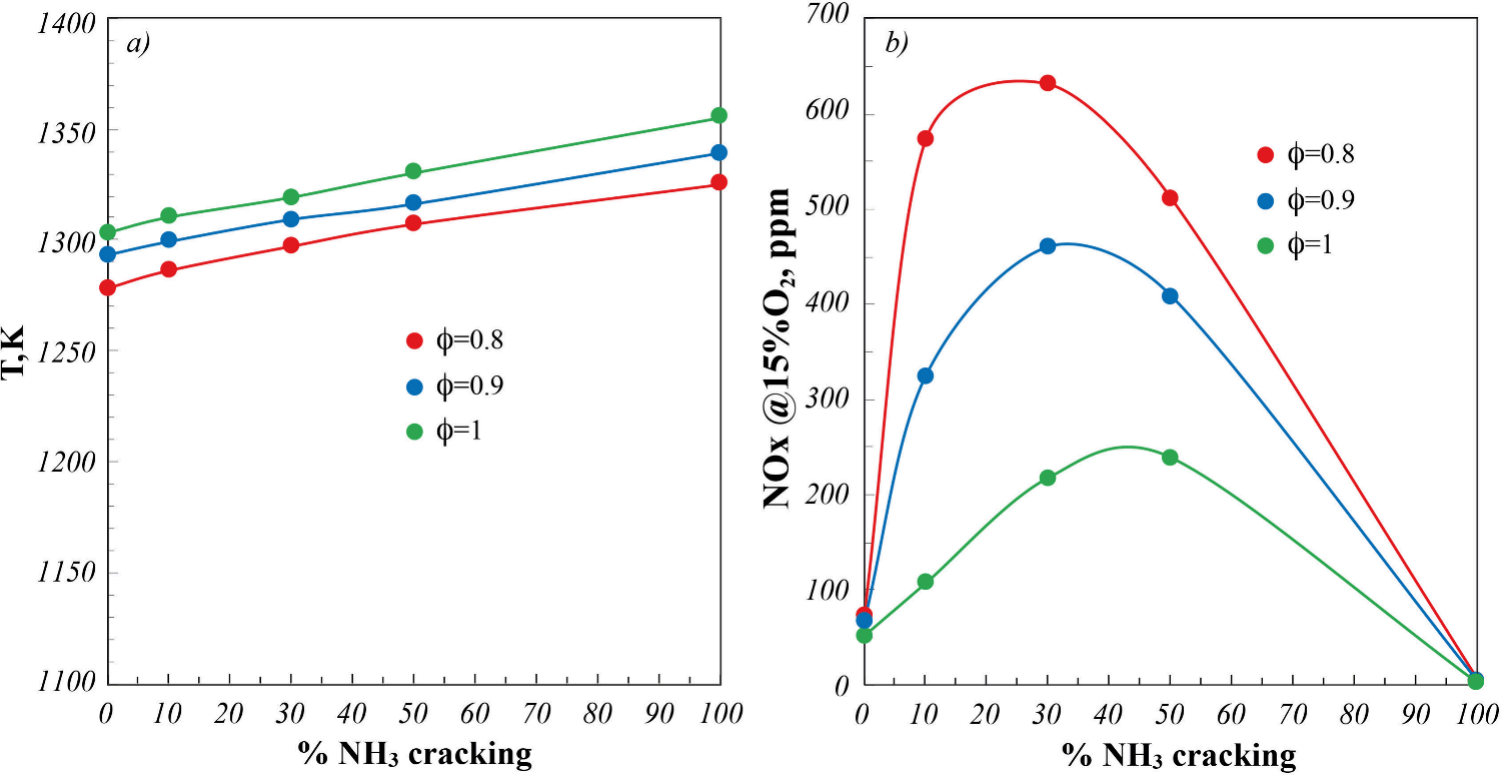
(a) Operating temperatures and (b) NO_*x*_ emissions for ϕ = 0.8–0.9–1 as a function of %NH_3_ cracking. *P* = 7 kW.

With respect to operating temperatures (Figure 11a), monotonic increasing trends are shown as a function of %NH_3_ cracking, as a result of the increasing %H_2_ of the fuel mixture, in agreement with results previously reported in Figure 7. However, the maximum detected temperature difference remains lower than 50 K. On the other hand, NO_*x*_ emissions (Figure 11b) exactly follow the same trend reported as a function of the %H_2_ in Figure 6 for NH_3_/H_2_ blends, testifying the effectiveness of MILD Combustion in flattening the influence of fuel mixture dilution by N_2_ derived from NH_3_ cracking, as a result of its intrinsic fuel flexibility.

## 5 Conclusions

The present study provides significant novel insights into the combustion behavior of NH_3_/H_2_ blends and cracked ammonia mixtures under MILD combustion conditions, systematically examining process stability, emissions speciation, and pollutant formation. Key findings are summarized in the following points:

- The obtained results showcase the remarkable fuel flexibility of the MILD process and the cyclonic flow reactor, allowing to achieve almost unchanged operating temperatures despite the significant change in the fuel composition. Specifically, temperatures are consistently below 1400 K, with a narrow variation of 100 K as the fuel shifts from pure NH_3_ to pure H_2_.
- Hydrogen addition substantially enhances NH_3_ combustion stability, significantly lowering the minimum operating temperature and expanding stability limits toward lower equivalence ratios.
- A key contribution is the demonstration that the dilution introduced by N_2_ from cracked ammonia mixtures does not adversely affect combustion stability or significantly alter emissions profiles under MILD conditions. This emphasizes the robustness and operational resilience of MILD combustion technology, particularly valuable for practical applications involving partially cracked ammonia mixtures.
- Peculiar NO*_x_* behaviors were observed in NH_3_/H_2_ blends, where blending leads to higher NO_*x*_ emissions than either pure NH_3_ or pure H_2_. This increase is due primarily to enhanced radical production by hydrogen addition, promoting NH_2_ oxidation to NO_*x*_. However, the intrinsically low-emission characteristics provided by MILD combustion consistently maintain NO_*x*_ levels below critical thresholds.
  Remarkably, pure hydrogen combustion under MILD conditions exhibited NOx emissions consistently below 40 ppm, significantly lower than traditional combustion systems, attributed to inherently moderate temperature conditions.

The results from this study affirm MILD combustion as a highly promising technology for the efficient and environmentally sustainable use of ammonia-based fuels, particularly emphasizing the capability to handle fuels derived from ammonia cracking without detrimental impacts on emissions or operational stability. The principal insights for engineers and practitioners are that MILD combustion reliably enables stable, efficient, and low-NO_*x*_ operation (<100 ppm) of NH_3_/H_2_ and partially cracked ammonia mixtures, addressing critical practical limitations associated with NH_3_ as a fuel.

Potential future research directions for this study involve further exploration of advanced fuel injection and mixing strategies within MILD combustion systems to potentially lower NO_*x*_ emissions even further, particularly for high hydrogen blends. Investigating techno-economic and lifecycle assessments for scale-up scenarios using cracked ammonia fuels under MILD conditions would be also valuable to facilitate broader industrial applications.

## Supplementary Material



## Notes

Section 1: Detailed information about the experimental conditions; Section 2: Reactants inlet velocities and reactor residence times; Section 3: Extinction conditions analysis; Section 4: Fuels oxidation chemistry and NO*_x_* formation routes; Section 5: Normalized NOx emissions to the 10%NH_3_ cracking dilution level; Section 6: CFD analyses for pure H_2_ and fully cracked NH_3_ mixture (PDF)
