# Supplementary material for: Ammonia/Hydrogen and Cracked Ammonia Combustion
Source: Energy Fuels. 2025 Sep 29;39(40):19512–25. doi: 10.1021/acs.energyfuels.5c02759 (PMC12519573; doi:10.1021/acs.energyfuels.5c02759)
Supplement: Supplementary file 1 [file ef5c02759_si_001.pdf]

Supplementary material

Ammonia/Hydrogen and cracked Ammonia Combustion

Giovani Battista Ariemma[a], Giancarlo Sorrentino[a,], Mara de Joannon[a], Raffaele Ragucci[a], Pino Sabia[a]*

a. Institute of Sciences and Technologies for Sustainable Energy and Mobility (STEMS-CNR), Naples, Italy

Section 1: Detailed information about the experimental conditions

In Table S1 the reactant mixture compositions, volumetric flow rates of fuel, air and the total one are reported for all the conditions experimentally investigated.

Table S1. Detailed experimental conditions in terms of reactants mixture composition and volumetric flow rates.

**100%Ammonia**							
ϕ	%H_2	%NH_3	%O_2	%N_2	Total flow rate ($x10^{-3}$), m^3/s	Air flow rate ($x10^{-3}$), m^3/s	Fuel flow rate ($x10^{-3}$), m^3/s
0.4		*10.08*	*18.89*	*71.03*	*5.39*	*4.85*	*0.544*
0.5		*12.29*	*18.43*	*69.29*	*4.42*	*3.88*	
0.6		*14.39*	*17.99*	*67.63*	*3.77*	*3.23*	
0.7		*16.39*	*17.56*	*66.04*	*3.31*	*2.77*	
0.8		*18.31*	*17.16*	*64.53*	*2.97*	*2.43*	
0.9		*20.13*	*16.78*	*63.09*	*2.70*	*2.16*	
1		*21.88*	*16.41*	*61.71*	*2.48*	*1.94*	
1.1		*23.55*	*16.06*	*60.39*	*2.30*	*1.76*	
1.2		*25.16*	*15.72*	*59.12*	*2.16*	*1.62*	
**5%Hydrogen**							
ϕ	%H_2	%NH_3	%O_2	%N_2	Total flow rate ($x10^{-3}$), m^3/s	Air flow rate ($x10^{-3}$), m^3/s	Fuel flow rate ($x10^{-3}$), m^3/s
0.4	*0.51*	*9.72*	*18.86*	*70.91*	*5.38*	*4.83*	*0.550*
0.5	*0.62*	*11.84*	*18.39*	*69.14*	*4.41*	*3.86*	
0.6	*0.73*	*13.87*	*17.94*	*67.46*	*3.77*	*3.22*	
0.7	*0.83*	*15.79*	*17.52*	*65.86*	*3.31*	*2.76*	
0.8	*0.93*	*17.63*	*17.11*	*64.33*	*2.96*	*2.41*	

0.9	1.02	19.39	16.72	62.87	2.70	2.15	
1	1.11	21.06	16.35	61.48	2.48	1.93	
1.1	1.19	22.67	16.00	60.15	2.31	1.76	
1.2	1.27	24.20	15.66	58.87	2.16	1.61	
**10%Hydrogen**							
ϕ	$\%H_2$	$\%NH_3$	$\%O_2$	$\%N_2$	Total flow rate ($x10^{-3}$), m^3/s	Air flow rate ($x10^{-3}$), m^3/s	Fuel flow rate ($x10^{-3}$), m^3/s
0.4	1.04	9.35	18.83	70.79	5.36	4.80	0.557
0.5	1.27	11.39	18.35	69.00	4.40	3.84	
0.6	1.48	13.33	17.90	67.29	3.76	3.20	
0.7	1.69	15.18	17.47	65.67	3.30	2.74	
0.8	1.88	16.94	17.05	64.13	2.96	2.40	
0.9	2.07	18.62	16.66	62.65	2.69	2.13	
1	2.25	20.22	16.29	61.24	2.48	1.92	
1.1	2.42	21.75	15.93	59.90	2.31	1.75	
1.2	2.58	23.22	15.59	58.61	2.16	1.60	
**20%Hydrogen**							
ϕ	$\%H_2$	$\%NH_3$	$\%O_2$	$\%N_2$	Total flow rate ($x10^{-3}$), m^3/s	Air flow rate ($x10^{-3}$), m^3/s	Fuel flow rate ($x10^{-3}$), m^3/s
0.4	2.14	8.57	18.76	70.53	5.32	4.75	0.571
0.5	2.61	10.44	18.27	68.68	4.37	3.80	
0.6	3.05	12.21	17.80	66.94	3.74	3.17	
0.7	3.47	13.89	17.36	65.28	3.29	2.72	
0.8	3.87	15.49	16.94	63.70	2.95	2.38	
0.9	4.25	17.01	16.54	62.19	2.68	2.11	
1	4.62	18.47	16.16	60.76	2.47	1.90	
1.1	4.96	19.86	15.79	59.39	2.30	1.73	
1.2	5.30	21.18	15.45	58.08	2.15	1.58	
**30%Hydrogen**							
ϕ	$\%H_2$	$\%NH_3$	$\%O_2$	$\%N_2$	Total flow rate ($x10^{-3}$), m^3/s	Air flow rate ($x10^{-3}$), m^3/s	Fuel flow rate ($x10^{-3}$), m^3/s
0.4	3.32	7.75	18.68	70.25	5.29	4.70	0.585
0.5	4.04	9.43	18.18	68.35	4.35	3.76	
0.6	4.72	11.01	17.70	66.56	3.72	3.13	
0.7	5.37	12.52	17.25	64.86	3.28	2.69	
0.8	5.98	13.95	16.82	63.24	2.94	2.35	
0.9	6.56	15.32	16.41	61.71	2.68	2.09	
1	7.12	16.62	16.02	60.24	2.47	1.88	
1.1	7.65	17.85	15.65	58.85	2.30	1.71	
1.2	8.16	19.03	15.30	57.51	2.16	1.57	
**40%Hydrogen**							
ϕ	$\%H_2$	$\%NH_3$	$\%O_2$	$\%N_2$	Total flow rate ($x10^{-3}$), m^3/s	Air flow rate ($x10^{-3}$), m^3/s	Fuel flow rate ($x10^{-3}$), m^3/s
0.4	4.58	6.87	18.60	69.95	5.24	4.64	0.600
0.5	5.56	8.35	18.09	68.00	4.32	3.72	
0.6	6.50	9.75	17.60	66.16	3.70	3.10	
0.7	7.38	11.07	17.13	64.42	3.25	2.65	
0.8	8.22	12.33	16.69	62.76	2.92	2.32	
0.9	9.01	13.52	16.27	61.19	2.66	2.06	
1	9.77	14.66	15.88	59.70	2.46	1.86	
1.1	10.49	15.74	15.50	58.27	2.29	1.69	
1.2	11.18	16.77	15.14	56.92	2.15	1.55	
**50%Hydrogen**							
ϕ	$\%H_2$	$\%NH_3$	$\%O_2$	$\%N_2$	Total flow rate ($x10^{-3}$), m^3/s	Air flow rate ($x10^{-3}$), m^3/s	Fuel flow rate ($x10^{-3}$), m^3/s
0.4	5.93	5.93	18.52	69.63	5.21	4.59	0.616

0.5	*7.19*	*7.19*	*17.99*	*67.63*	*4.29*	*3.67*	
0.6	*8.39*	*8.39*	*17.48*	*65.73*	*3.68*	*3.06*	
0.7	*9.52*	*9.52*	*17.01*	*63.95*	*3.24*	*2.62*	
0.8	*10.60*	*10.60*	*16.56*	*62.25*	*2.91*	*2.29*	
0.9	*11.61*	*11.61*	*16.13*	*60.65*	*2.66*	*2.04*	
1	*12.58*	*12.58*	*15.72*	*59.12*	*2.45*	*1.83*	
1.1	*13.50*	*13.50*	*15.34*	*57.67*	*2.29*	*1.67*	
1.2	*14.37*	*14.37*	*14.97*	*56.29*	*2.15*	*1.53*	
**60%Hydrogen**							
ϕ	%H_2	%NH_3	%O_2	%N_2	Total flow rate ($x10^{-3}$), m^3/s	Air flow rate ($x10^{-3}$), m^3/s	Fuel flow rate ($x10^{-3}$), m^3/s
0.4	*7.37*	*4.91*	*18.43*	*69.29*	*5.15*	*4.52*	
0.5	*8.94*	*5.96*	*17.88*	*67.22*	*4.25*	*3.62*	
0.6	*10.42*	*6.94*	*17.36*	*65.28*	*3.65*	*3.02*	
0.7	*11.81*	*7.87*	*16.87*	*63.44*	*3.21*	*2.58*	
0.8	*13.13*	*8.75*	*16.41*	*61.71*	*2.89*	*2.26*	*0.633*
0.9	*14.38*	*9.58*	*15.97*	*60.06*	*2.64*	*2.01*	
1	*15.56*	*10.37*	*15.56*	*58.51*	*2.44*	*1.81*	
1.1	*16.68*	*11.12*	*15.17*	*57.03*	*2.27*	*1.64*	
1.2	*17.75*	*11.83*	*14.79*	*55.62*	*2.14*	*1.51*	
**70%Hydrogen**							
ϕ	%H_2	%NH_3	%O_2	%N_2	Total flow rate ($x10^{-3}$), m^3/s	Air flow rate ($x10^{-3}$), m^3/s	Fuel flow rate ($x10^{-3}$), m^3/s
0.4	*8.93*	*3.83*	*18.33*	*68.92*	*5.11*	*4.46*	
0.5	*10.81*	*4.63*	*17.76*	*66.79*	*4.22*	*3.57*	
0.6	*12.59*	*5.39*	*17.23*	*64.79*	*3.62*	*2.97*	
0.7	*14.26*	*6.11*	*16.73*	*62.90*	*3.20*	*2.55*	
0.8	*15.83*	*6.79*	*16.26*	*61.13*	*2.88*	*2.23*	*0.651*
0.9	*17.32*	*7.42*	*15.81*	*59.44*	*2.63*	*1.98*	
1	*18.73*	*8.03*	*15.39*	*57.85*	*2.43*	*1.78*	
1.1	*20.07*	*8.60*	*14.99*	*56.35*	*2.27*	*1.62*	
1.2	*21.34*	*9.14*	*14.61*	*54.91*	*2.14*	*1.49*	
**80%Hydrogen**							
ϕ	%H_2	%NH_3	%O_2	%N_2	Total flow rate ($x10^{-3}$), m^3/s	Air flow rate ($x10^{-3}$), m^3/s	Fuel flow rate ($x10^{-3}$), m^3/s
0.4	*10.60*	*2.65*	*18.22*	*68.52*	*5.06*	*4.39*	
0.5	*12.83*	*3.21*	*17.64*	*66.32*	*4.18*	*3.51*	
0.6	*14.92*	*3.73*	*17.09*	*64.26*	*3.60*	*2.93*	
0.7	*16.88*	*4.22*	*16.58*	*62.33*	*3.18*	*2.51*	
0.8	*18.72*	*4.68*	*16.09*	*60.50*	*2.86*	*2.19*	*0.670*
0.9	*20.47*	*5.12*	*15.63*	*58.78*	*2.62*	*1.95*	
1	*22.11*	*5.53*	*15.20*	*57.16*	*2.43*	*1.76*	
1.1	*23.67*	*5.92*	*14.79*	*55.62*	*2.27*	*1.60*	
1.2	*25.14*	*6.29*	*14.41*	*54.16*	*2.13*	*1.46*	
**90%Hydrogen**							
ϕ	%H_2	%NH_3	%O_2	%N_2	Total flow rate ($x10^{-3}$), m^3/s	Air flow rate ($x10^{-3}$), m^3/s	Fuel flow rate ($x10^{-3}$), m^3/s
0.4	*12.42*	*1.38*	*18.11*	*68.09*	*5.00*	*4.31*	
0.5	*15.01*	*1.67*	*17.51*	*65.82*	*4.14*	*3.45*	
0.6	*17.42*	*1.94*	*16.94*	*63.70*	*3.57*	*2.88*	
0.7	*19.69*	*2.19*	*16.41*	*61.71*	*3.16*	*2.47*	
0.8	*21.82*	*2.42*	*15.91*	*59.84*	*2.85*	*2.16*	*0.691*
0.9	*23.83*	*2.65*	*15.45*	*58.08*	*2.61*	*1.92*	
1	*25.72*	*2.86*	*15.00*	*56.42*	*2.42*	*1.73*	
1.1	*27.51*	*3.06*	*14.59*	*54.85*	*2.26*	*1.57*	
1.2	*29.20*	*3.24*	*14.19*	*53.37*	*2.13*	*1.44*	

95%Hydrogen							
ϕ	%H_2	%NH_3	%O_2	%N_2	Total flow rate ($x10^{-3}$), m^3/s	Air flow rate ($x10^{-3}$), m^3/s	Fuel flow rate ($x10^{-3}$), m^3/s
0.4	*13.38*	*0.70*	*18.05*	*67.86*	*4.98*	*4.28*	*0.701*
0.5	*16.16*	*0.85*	*17.43*	*65.56*	*4.12*	*3.42*	
0.6	*18.75*	*0.99*	*16.86*	*63.40*	*3.55*	*2.85*	
0.7	*21.18*	*1.11*	*16.32*	*61.38*	*3.14*	*2.44*	
0.8	*23.46*	*1.23*	*15.82*	*59.48*	*2.84*	*2.14*	
0.9	*25.60*	*1.35*	*15.35*	*57.70*	*2.60*	*1.90*	
1	*27.62*	*1.45*	*14.90*	*56.03*	*2.41*	*1.71*	
1.1	*29.52*	*1.55*	*14.48*	*54.44*	*2.25*	*1.55*	
1.2	*31.32*	*1.65*	*14.08*	*52.95*	*2.13*	*1.43*	
**100%Hydrogen**							
ϕ	%H_2	%NH_3	%O_2	%N_2	Total flow rate ($x10^{-3}$), m^3/s	Air flow rate ($x10^{-3}$), m^3/s	Fuel flow rate ($x10^{-3}$), m^3/s
0.4	*14.39*		*17.99*	*67.63*	*4.95*	*4.24*	*0.712*
0.5	*17.36*		*17.36*	*65.28*	*4.10*	*3.39*	
0.6	*20.13*		*16.78*	*63.09*	*3.53*	*2.82*	
0.7	*22.73*		*16.23*	*61.04*	*3.13*	*2.42*	
0.8	*25.16*		*15.72*	*59.12*	*2.83*	*2.12*	
0.9	*27.44*		*15.24*	*57.32*	*2.59*	*1.88*	
1	*29.59*		*14.79*	*55.62*	*2.40*	*1.69*	
1.1	*31.61*		*14.37*	*54.02*	*2.25*	*1.54*	
1.2	*33.52*		*13.97*	*52.51*	*2.12*	*1.41*	

Section 2: Reactants inlet velocities and reactor residence times

In Figure S1 the fuel (a) and oxidizer (b) inlet velocities, along with the characteristic residence times (c) obtained by scaling the mean residence time computed in non-reactive conditions with the average temperature in the reactor, are shown as a function of the inlet equivalence ratio (ϕ) and NH_3/H_2 mixture composition.

In particular, fuel velocities (Figure S1a) increase from about 160 m/s for the pure NH_3 case up to about 200 m/s for pure H_2 one, due to the imposed conditions of constant thermal power (7 kW) and the lower volumetric heating value of H_2. Instead, oxidizer velocities (Figure S1b) slightly decrease by moving from pure NH_3 to pure H_2 for a fixed equivalence ratio, with a maximum difference always lowers than 12% with respect to the pure NH_3 reference case. Such slightly differences of the inlet reactants velocities very slightly affect the velocity field and residence times distribution within the reactor, as testified by the completely overlapping characteristic residence times (Figure S1c) for all the investigated NH_3/H_2 mixtures.

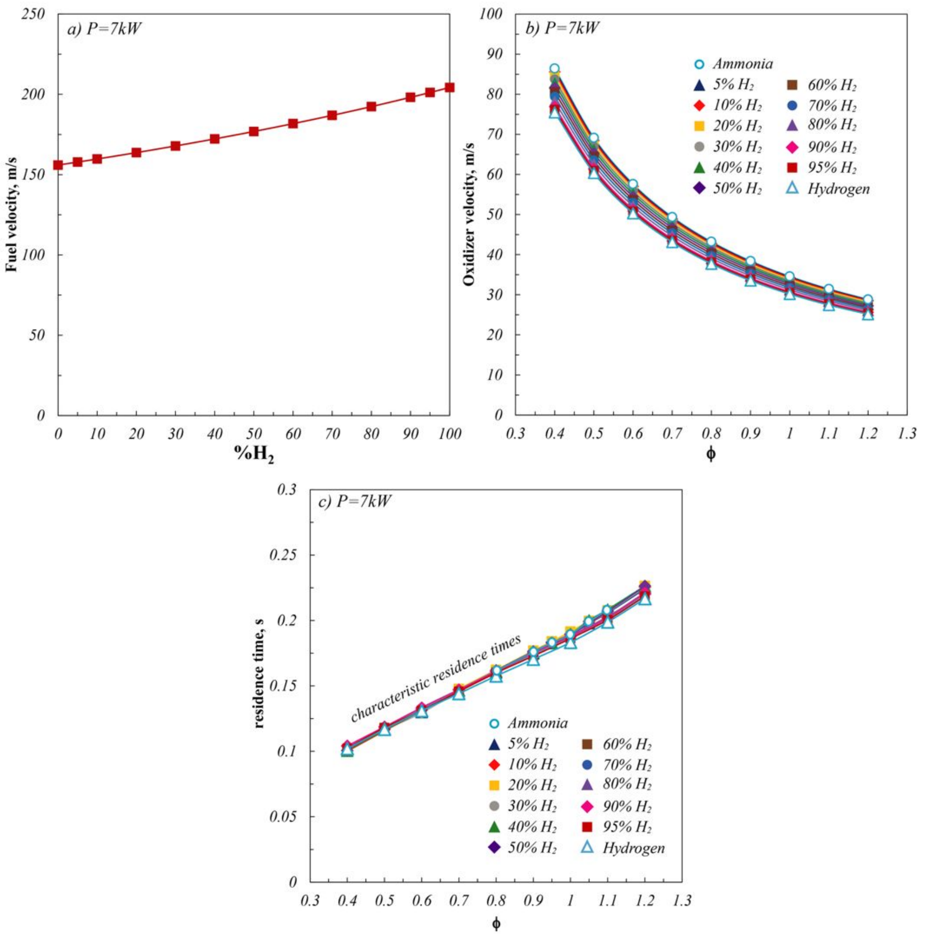

Figure S1*. Fuel (a) and oxidizer (b) inlet velocities, characteristic residence times (c) as a function of the inlet equivalence ratio (ϕ) and NH_3/H_2 mixture composition. P=7 kW.*

Section 3: Extinction conditions analysis

In this section, the interplay between chemical and residence times in determining the approaching extinction conditions in the cyclonic burner was analysed. In this respect, ignition delay times of fuel blends for which extinction phenomena were experimentally detected, i.e. pure NH_3 case and NH_3/H_2 blends up to 20%H_2, were evaluated. In particular, the computed minimum temperature allowing the reactant mixture ignition in the experimental residence time range (0.1s<τ<0.15s) was compared with the experimental temperature detected in the mixing region among fuel, oxidizer and recirculating combustion products ($T_{lateral}$), where first reactive kernels form and develop.

In this respect, ignition delay times were computed by the Closed Homogeneous Batch Reactor code of the CHEMKIN PRO[1] package, with the updated detailed kinetic mechanism for NH_3/H_2 mixtures of Shrestha et al.[2]. In particular, $NH_3/H_2/O_2/N_2$ reactant mixtures were considered, by fixing the dilution level (%N_2 v/v) equal to 90%, in agreement with the typical average local dilution levels characterizing the reactants mixing region of the cyclonic burner. The system was simulated as adiabatic, in the operating temperature range 800K<T<1500K and in the equivalence ratio range (0.4<ϕ<0.8). Specifically, a temperature increment of 10K

with respect to the non-reactive conditions was considered as identification criterium of the mixture ignition.

In this respect, in Figure S2a ignition delay times monotonically decrease as a function of the operating temperature (T) and the volumetric hydrogen concentration (%H_2) in the considered fuel mixture, as expected. In addition, ignition delay times show a very slight dependence on the equivalence ratio (ϕ), with almost overlapping profiles for each considered fuel mixture. In particular, by considering the effective residence time range characterizing the cyclonic reactor for the considered fuel mixtures ($0.1s<\tau<0.15s$), the corresponding minimum ignition temperature for the pure NH_3 case is about 1200 K, while for fuel mixtures with $5<\%H_2<20$ it ranges between $950K<T<1000K$.

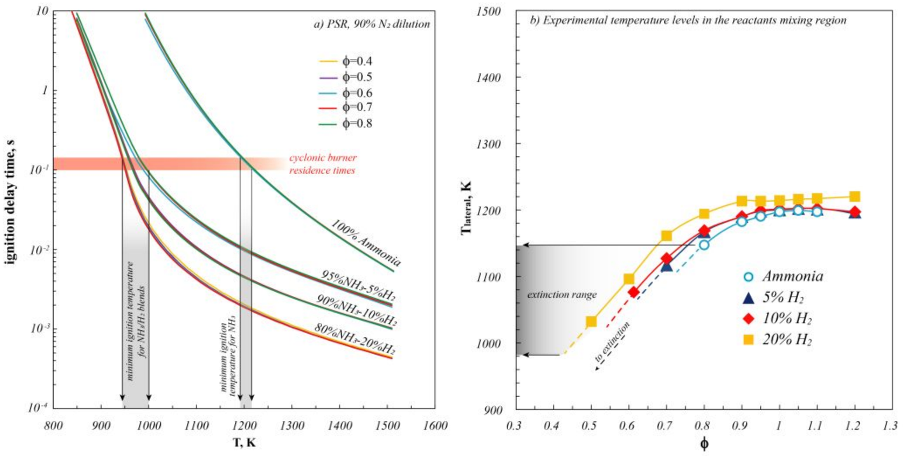

Figure S2. Ignition delay time (a) and experimental temperatures levels in the reactants mixing region (b) for mixtures approaching experimental extinction conditions. P=7kW.

On the other hand, the $T_{lateral}$ profiles detected in the reactants mixing region (Figure S2b) show a monotonic increasing trend as a function of the equivalence ratio (ϕ), for all the considered cases. In particular, it is worth stressing that extinction conditions (dashed lines) are detected for temperature levels ($970K<T_{lateral}<1150K$) approaching the minimum one required for the mixture ignition (Figure S2a). As a result, this condition causes the combustion process stabilization becomes very sensitive to the competition between the characteristic chemical and residence times.

Section 4: Fuels oxidation chemistry and NOx formation routes

In order to highlight main kinetic pathways involved in the NOx production and consumption entailing the marked NOx emissions increase detected by blending NH_3 with H_2 even at low %H_2, Reaction Rate and flux diagrams analyses were performed. In particular, the system was simulated as isothermal PSR, with a volume equal to the cyclonic flow burner one (2000 cm^3), at three different temperature levels, i.e. T=1200-1300-1400 K, chosen as representative of the average experimental temperature levels detected for all the investigated NH_3/H_2 blends. A fuel mixture at ϕ=0.8 was considered as refence case, conditions in which the maximum NOx

increase was experimentally detected with respect to the pure ammonia case. The updated detailed kinetic mechanism for NH_3/H_2 blends of Shrestha et al. [2] was used.

First, flux diagrams were realized for 90%NH_3-10%H_2 and 10%NH_3-90%H_2 fuel mixtures, chosen as limit reference cases of the investigated conditions. The main species and the relative pathways are reported with a different colour code: ammonia and its intermediate species are reported in black, hydrogen and its intermediate species in red as the main radical species that support the branching mechanism. In addition, the main species and reactions directly involved in the NOx formation/consumption are highlighted in green. The same colour code is used for the arrows of reactions in which these species are involved in. The arrow thickness is proportional to the reaction rate values, reported in the round brackets, whose order of magnitude is reported in the legend.

In this respect, Figure S3 shows the main kinetic pathways characterizing the oxidation of 90%NH_3-10%H_2 fuel mixture, for T=1300 K and ϕ=0.8.

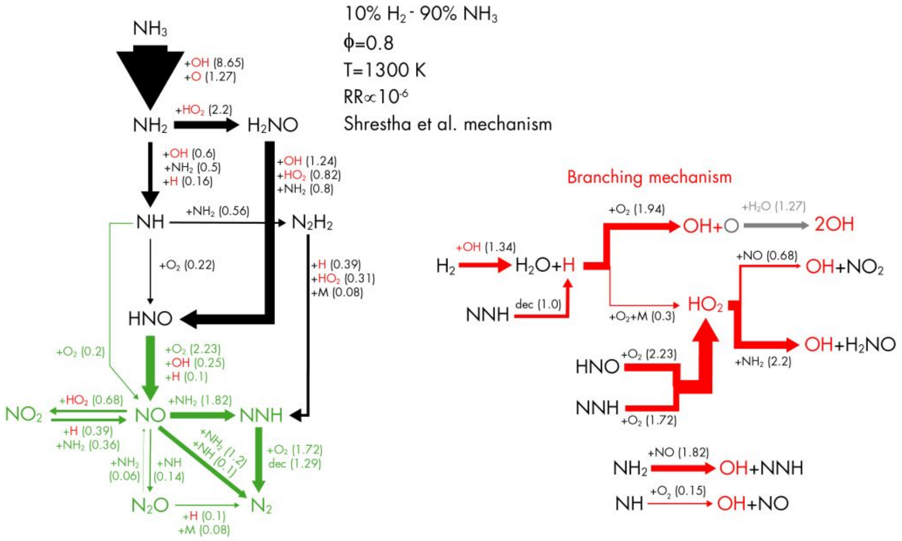

Figure S3. Flux diagrams for 90%NH_3-10%H_2. T=1300K. ϕ=0.8. Shrestha et al. mechanism.

NH_3 follows the pathway $NH_3 \rightarrow NH_2 \rightarrow H_2NO \rightarrow HNO \rightarrow NO \rightarrow N_2$. In particular, NH_3 is converted to NH_2 by OH radicals, and secondarily by O radicals. Then, NH_2 radicals are mainly converted to H_2NO by HO_2 radicals, while to NH by OH, NH_2 and O radicals. Then, H_2NO is rapidly converted to HNO by OH and HO_2 radicals. On the other hand, NH is mainly converted to HNO and N_2H_2 by O_2 and NH_2 radical, respectively. In addition, NH also directly forms NO through the reaction $NH+O_2=NO+OH$. This latter, together with the set of HNO consumption reactions by O_2, OH and H radicals are the main responsible of NO production.

On the other hand, NO is mainly converted to N_2 by the typical DeNOx chemistry, through NH_2 and NH species, and it is also involved in the NO-NO_2 loop with a set of reactions that lead to the production of NO_2 through HO_2 radicals, and its conversion back to NO with H and NH_2 radicals. It is worth stressing that the thermal NOx routes are not active while the thermal DeNOx process is active and comparable to the main reactions involved in the NO production, as expected in the temperatures range of MILD Combustion (T<1600K).

On the other hand, the branching mechanism is controlled by the H_2 chemistry. In particular, reaction $H+O_2=OH+O$ and, consequently, $H_2O+O=2OH$ are the main ones involved in OH production. NH_2 and NH radicals also contribute to OH production. In addition, HO_2 radicals are mainly formed through the reactions $HNO+O_2=HO_2+NO$ and $NNH+O_2=NHO_2+N_2$. HO_2 radicals also produce OH radicals by reacting with NH_2 and NO, respectively.

Coherent results were obtained for the 10%NH_3-90%H_2 fuel mixture, shown in Figure S4.

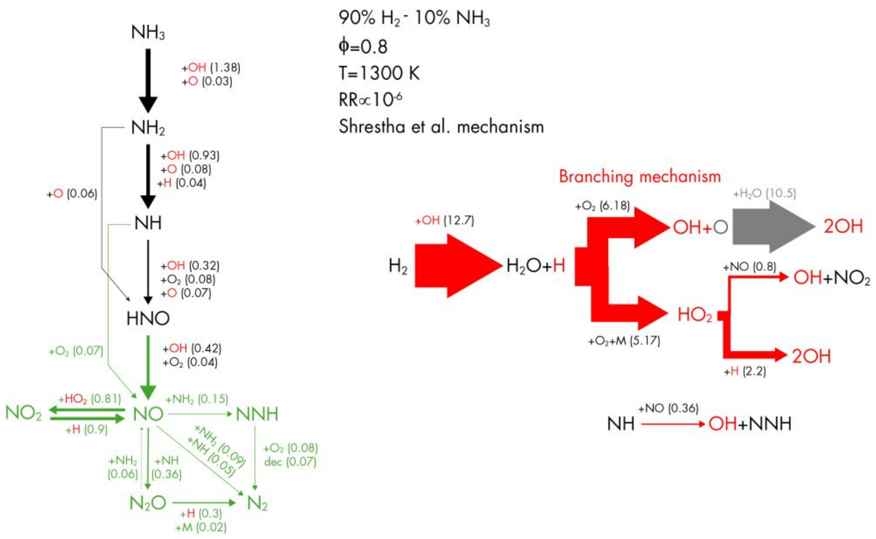

Figure S4. Flux diagrams for 10%NH_3-90%H_2. T=1300K. ϕ=0.8. Shrestha et al. mechanism.

In this respect, NH_3 oxidation follows the pathway $NH_3 \rightarrow NH_2 \rightarrow NH \rightarrow HNO \rightarrow NO \rightarrow N_2$, with the branching mechanism totally controlled by the H_2 chemistry. A slight difference with respect to the 90%NH_3-10%H_2 case is related to the conversion of NH_2, that is mainly converted to NH at high %H_2 instead of H_2NO, and NH species not going through the N_2H_2 route. As in the 90%NH_3-10%H_2, by increasing the %H_2 in the fuel mixture the NH_3 oxidation is always sustained by the high temperature branching reactions of the H_2/O_2 subsystem, that is entirely responsible for the production of OH, H and HO_2 radicals. In general, the increase of %H_2 in the fuel blend provide a more evident pool of radical species production, as highlighted by the red arrow thickness and the reaction rate values shown in the flux diagram.

It is worth to stress that results reported in Figure S3 and Figure S4 has a general validity with respect to the operating temperature (in the range 1200-1400 K, coherent with the experimental temperature levels) and fuel mixtures with different %H_2 and NH_3 cracking levels, whose numerical results are not shown for the sake of brevity.

In particular, the analyzed reaction pathways show the NO production is always due to the NH_3 oxidation scheme, independently of the considered NH_3/H_2 blend. In particular, they highlight the key role of NH_2, that is the main one involved in both the NO production and consumption, and OH, that is the main species sustaining the NH_3 oxidation and its conversion to NO.

Furthermore, analyzed reaction pathways clearly show the direct effect of H_2 on the radical pool production. Specifically, with reference to the reported branching mechanism, H radical is the first species involved in. For high %NH_3, this is mainly produced by H_2+OH reaction

and NNH decomposition (NNH=N_2+H). By increasing the %H_2, the H radicals' production suddenly increases since supported by the H_2 oxidation chemistry, that is totally involved in the system branching mechanism. In fact, the main H_2 consumption reaction H_2+OH=H_2O+H directly and rapidly produces H radicals that boost the OH and HO_2 radicals' production. As result of the boosted radical pool production, the NH_2 availability for NO consumption through DeNOx reactions decreases, due to its increased conversion to NH and HNO due to the higher OH availability. This behaviour entails sudden NOx emissions increase with respect to the pure NH_3, even at low %H_2 in the fuel mixture, as testified by the experimental results of Figure 4 and Figure 6.

Afterwards, in order to highlight the main parameters affecting the NOx distribution with respect to the NH_3/H_2 fuel mixture composition, Reaction Rate analyses were performed. In particular, the reaction rates of the main reactions involved in NO production and consumption previously identified were analysed as a function of the %H_2 in the fuel blend.

In this respect, in Figure S5 Reaction Rates are reported for T=1300 K and ϕ=0.8, in agreement with the flux diagrams shown in Figure S3 and Figure S4.

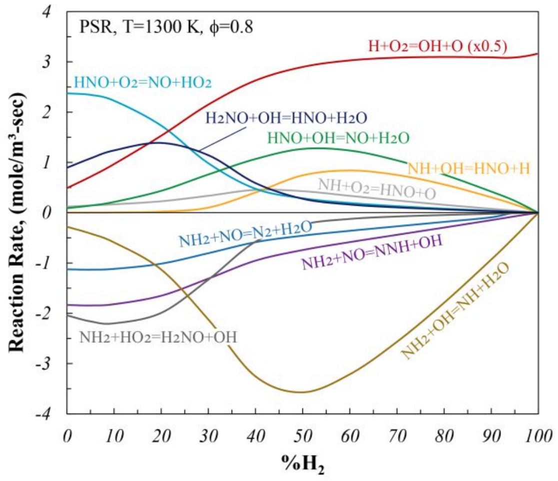

Figure S5. Reaction rate of the main NOx formation and consumption reactions as a function of the %H_2 in the fuel mixture. T=1300K. ϕ=0.8. Shrestha et al. mechanism.

The OH production through the H+O_2=OH+O reaction shows a monotonic increasing trend as a function of %H_2, as expected due to the increasing H_2 concentration in the fuel mixtures. Instead, the main reactions involving the HNO conversion to NO show different trends. In particular, the HNO+OH=NO+H_2O reaction shows a non-monotonic trend, with a maximum localized between 50<%H_2<60. This latter is due to the different HNO and OH availability dependently on the %H_2 in the fuel mixture. In fact, for %H_2<50-60 the OH production increase by increasing the %H_2 play the major role in entailing increasing HNO conversion to NO, although the progressively lower HNO concentration. Conversely, for %H_2>50-60 the always higher OH production does not compensate the further decreasing HNO availability, thus entailing a decreasing trend for the HNO+OH=NO+H_2O reaction.

Similar non-monotonic trends characterize the HNO formation reactions $NH+O_2=HNO+O$ and $NH+OH=HNO+H$, induced by a similar competition between NH and OH radicals availability as a function of $\%H_2$, and the HNO formation through $H_2NO+OH=HNO+H_2O$ reaction. This latter increases up to $\%H_2=20\%$ and then decreases for higher $\%H_2$. This reaction, in particular, increases the HNO availability for low $\%H_2$, thus entailing the $HNO+O_2=NO+HO_2$ slightly increases up to $\%H_2=10$ and then decreases for higher $\%H_2$.

With respect to the NO consumption, DeNOx reactions $NH_2+NO=NNH+OH$ and $NH_2+NO=N_2+H_2O$ show decreasing profiles with $\%H_2$, due to the decreasing NH_2 availability by increasing $\%H_2$. In fact, NH_2 is mainly converted to NH through $NH_2+OH=NH+H_2O$ reaction. This increases up to about $\%H_2=50$ as result of the increasing OH production and sufficient NH_2 availability, while decreasing for higher $\%H_2$ due to the always lower NH_2 concentration. Furthermore, NH_2 is also converted to H_2NO by HO_2 radicals through $NH_2+HO_2=H_2NO+OH$ reaction, that is the main one sustaining the H_2NO formation.

The analyzed profiles of the main reactions involved in the NO production and consumption highlight the peculiar interplay between the NH_2 and HNO radicals, deriving from the NH_3 oxidation pathway, and the OH radicals, whose formation is mainly supported by the H_2 oxidation. In particular, their different availability in dependence on the fuel mixture composition ($\%H_2$) entails non-monotonic NOx emissions as a function of the $\%H_2$, thus explaining the non-monotonic NOx emissions trend experimentally detected for NH_3/H_2 blends and cracked NH_3 mixtures shown in Figure 6 and Figure 11 of the paper.

Section 5: Normalized NOx emissions to the 10%NH_3 cracking dilution level

In order to isolate the effects of fuel composition (i.e. NH_3/H_2 ratio and associated cracking) from those due to changes in the inlet dilution, the NOx emissions trends showed in Figure 7b and Figure 11b of the paper, were compared with the ones obtained by normalizing the detected NOx emissions at the same value of the inlet dilution condition, for each NH_3 cracking ratio. In particular, the inlet dilution level of the 10%NH_3 cracking was selected as reference, while the results shown in Figure S6.

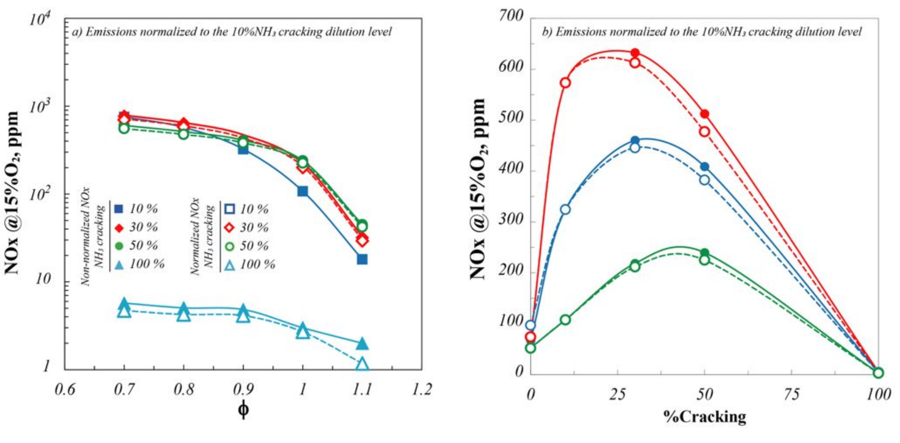

Figure S6. Normalized NOx emissions to the 10%NH_3 cracking dilution level as a function of ϕ (a) and %Cracking (b).

In particular, Figure S6a and Figure S6b clearly highlight that the normalized NOx trends with respect to ϕ and %Cracking overlap with those obtaining without normalization, testifying that negligible impact of the different inlet dilution levels of the investigated NH_3 cracking mixtures. In fact, such comparable trends confirm that the internal dilution effects induced by the cyclonic motion largely mitigate the effect of variations in inlet dilution.

Section 6: CFD analyses for pure H_2 and fully cracked NH_3 mixture

In order to better highlight the different temperature distribution and the absence of hot-spot regions induced by the partial H_2 dilution derived from fully cracked NH_3 mixtures (i.e. 75%H_2-25%N_2) with respect to the pure H_2 case, dedicated CDF simulations were performed.

In particular, the numerical modelling approach validated in our previous work [3] was used. Briefly, the 3D computational domain of the cyclonic burner was built by implementing only half of the total reactor volume, by taking advantage of the rotational periodicity resulting from the antisymmetric configuration of the burner geometry, thus applying a periodic boundary condition with rotation of 180°. A computational grid consisting of about 300 k grid elements was identified as suitable for the reference cases, with refinements selectively implemented for the reactants inlet regions and close the reactor walls. Boundary conditions were defined in agreement with experimental conditions for pure H_2 and fully cracked NH_3 mixture (i.e. 75%H_2-25%N_2), in terms of inlet velocity magnitude (corresponding to a thermal power input of 7kW and ϕ=1), temperature and composition of fuel and air. Furthermore, the heat exchange process through the reactor walls was handled as convective heat transfer, fixing the environment temperature equal to 500 K (experimental value). Then, the heat transfer coefficient was progressively modified in the range 60-70 W/m^2-K until matching the exhaust gas temperatures experimentally detected at the burner exit.

RANS simulations were performed by the commercial code ANSYS Fluent [4], employing the Reynolds Stress Model (RSM) [4] and assuming incompressible and ideal gas conditions, while the Enhanced Wall Treatment (EWT) [4] was adopted as near-wall modelling method. Second order upwind interpolation scheme was employed for the pressure-velocity coupling. Furthermore, radiative transport was handled by the Discrete Ordinate (DO) method with the weighted-sum-of-grey-gas (WSGG) [5] approach.

The Partially Stirred Reactor (PaSR) combustion model [6], in its static formulation with a mixing constant (C_{mix}) equal to 0.5 [3], was used to handle the turbulence-chemistry interaction, since very effective for MILD combustion conditions. Finally, the updated detailed kinetic mechanism for NH_3/H_2 mixtures of Shrestha et al. [2] was used as kinetic scheme.

Results are reported in Figure S7 in terms of temperature fields for the reference cases, evaluated on the reactor midplane chosen as representative of the whole reactor volume. In particular, for the pure H_2 case (Figure S7a) the whole reactor is characterized by higher temperatures than the fully cracked NH_3 mixture (Figure S7b). This is mainly ascribable to the influence of the inlet N_2 dilution imposed by the NH_3 cracking mixture, that has both a thermal and chemical influence. In fact, although the operating temperatures detected for the two

reference cases at the exit section of the cyclonic burner show differences lower than 50 K, testifying the limited thermal influence of the diluting N_2, their distribution within the combustion chamber is more markedly affected by the inlet fuel dilution. In fact, the inlet N_2 characterizing the fully cracked NH_3 mixture (75%H_2-25%N_2) entails longer chemical times than the pure H_2 case, entailing a more distributed heat release within the reactor. This behaviour prevents the formation of hot-spot regions and ensures a more uniform temperature distribution, thus explaining the experimental evidence obtained with respect to the pure H_2 and 75%H_2-25%N_2 fuel mixture.

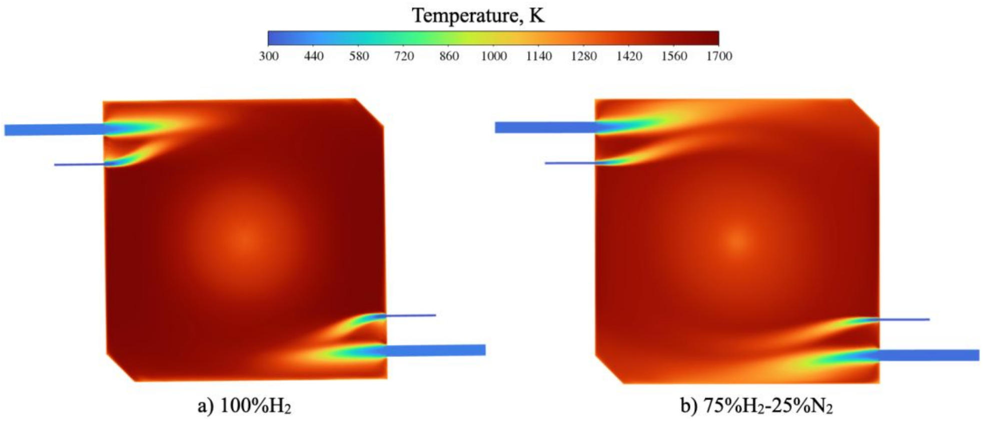

Figure S7. Temperature field on the reactor midplane 100%H_2 (a) and 75%H_2-25%N_2 (b) fuel mixture. P=7 kW. ϕ=1.

References

(1) ANSYS. CHEMKIN-PRO 15131. Reaction Design: San Diego 2013.

(2) Shrestha, K. P.; Lhuillier, C.; Barbosa, A. A.; Brequigny, P.; Contino, F.; Mounaïm-Rousselle, C.; Seidel, L.; Mauss, F. An Experimental and Modeling Study of Ammonia with Enriched Oxygen Content and Ammonia/Hydrogen Laminar Flame Speed at Elevated Pressure and Temperature. *Proceedings of the Combustion Institute* **2021**, *38* (2), 2163–2174. https://doi.org/10.1016/j.proci.2020.06.197.

(3) Ariemma, G. B.; Sorrentino, G.; Sabia, P.; Parente, A.; Ragucci, R.; de Joannon, M. Timescales Distribution and Reactive Structures in MILD Reactors for Different Energy Carriers. *Results in Engineering* **2024**, *24*, 103398. https://doi.org/10.1016/J.RINENG.2024.103398.

(4) ANSYS. Fluent ANSYS. ANSYS Fluent Theory Guide 19.1. Canonsburg 2019.

(5) Smith, T. F.; Shen, Z. F.; Friedman, J. N. *Evaluation of Coefficients for the Weighted Sum of Gray Gases Model*; 1982. http://heattransfer.asmedigitalcollection.asme.org/.

(6) Péquin, A.; Evans, M. J.; Chinnici, A.; Medwell, P. R.; Parente, A. The Reactor-Based Perspective on Finite-Rate Chemistry in Turbulent Reacting Flows: A Review from

Traditional to Low-Emission Combustion. *Applications in Energy and Combustion Science* **2023**, *16*, 100201. https://doi.org/10.1016/J.JAECS.2023.100201.
